# Bacterial extracellular vesicles as intranasal postbiotics: Detailed characterization and interaction with airway cells

**DOI:** 10.1002/jev2.70004

**Published:** 2024-10-21

**Authors:** Agnieszka Razim, Agnieszka Zabłocka, Anna Schmid, Michael Thaler, Viktor Černý, Tamara Weinmayer, Bradley Whitehead, Anke Martens, Magdalena Skalska, Mattia Morandi, Katy Schmidt, Magdalena E. Wysmołek, Akos Végvári, Dagmar Srutkova, Martin Schwarzer, Lukas Neuninger, Peter Nejsum, Jiri Hrdý, Johan Palmfeldt, Marco Brucale, Francesco Valle, Sabina Górska, Lukas Wisgrill, Aleksandra Inic‐Kanada, Ursula Wiedermann, Irma Schabussova

**Affiliations:** ^1^ Institute of Specific Prophylaxis and Tropical Medicine, Centre for Pathophysiology Infectiology and Immunology, Medical University of Vienna Vienna Austria; ^2^ Hirszfeld Institute of Immunology and Experimental Therapy Polish Academy of Sciences Wroclaw Poland; ^3^ Department of Infectious Diseases Aarhus University Hospital Aarhus Denmark; ^4^ Department of Clinical Medicine Aarhus University Aarhus Denmark; ^5^ Division of Neonatology, Paediatric Intensive Care and Neuropediatric, Department of Paediatrics and Adolescent Medicine, Comprehensive Centre for Paediatrics Medical University of Vienna Vienna Austria; ^6^ Department of Medical Physics, M. Smoluchowski Institute of Physics, Faculty of Physics Astronomy and Applied Computer Science, Jagiellonian University Krakow Poland; ^7^ Institute of Organic Chemistry and Biochemistry of the Czech Academy of Science Prague Czech Republic; ^8^ Research Support Facilities, Imaging Unit CIUS, Faculty of Life Sciences University of Vienna Vienna Austria; ^9^ Proteomics Biomedicum, Division of Chemistry I, Department of Medical Biochemistry and Biophysics Karolinska Institutet Stockholm Sweden; ^10^ Division of Chemistry I, Department of Medical Biochemistry and Biophysics Karolinska Institutet Stockholm Sweden; ^11^ Laboratory of Gnotobiology Institute of Microbiology of the Czech Academy of Sciences Novy Hradek Czech Republic; ^12^ Institute of Immunology and Microbiology, First Faculty of Medicine Charles University and General University Hospital Prague Czech Republic; ^13^ Research Unit for Molecular Medicine, Department of Clinical Medicine Aarhus University Aarhus Denmark; ^14^ Institute of Nanostructured Materials CNR‐ISMN Bologna Italy

**Keywords:** bacterial extracellular vesicles, Ec083, EVs, macrophage, NF‐κΒ signalling, nitric oxide, postbiotics, probiotic

## Abstract

*Escherichia coli* A0 34/86 (EcO83) is a probiotic strain used in newborns to prevent nosocomial infections and diarrhoea. This bacterium stimulates both pro‐ and anti‐inflammatory cytokine production and its intranasal administration reduces allergic airway inflammation in mice. Despite its benefits, there are concerns about the use of live probiotic bacteria due to potential systemic infections and gene transfer. Extracellular vesicles (EVs) derived from EcO83 (EcO83‐EVs) might offer a safer alternative to live bacteria. This study characterizes EcO83‐EVs and investigates their interaction with host cells, highlighting their potential as postbiotic therapeutics. EcO83‐EVs were isolated, purified, and characterised following the Minimal Information of Studies of Extracellular Vesicles (MISEV) guidelines. Ex vivo studies conducted in human nasal epithelial cells showed that EcO83‐EVs increased the expression of proteins linked to oxidative stress and inflammation, indicating an effective interaction between EVs and the host cells. Further in vivo studies in mice demonstrated that EcO83‐EVs interact with nasal‐associated lymphoid tissue, are internalised by airway macrophages, and stimulate neutrophil recruitment in the lung. Mechanistically, EcO83‐EVs activate the NF‐κΒ signalling pathway, resulting in the nitric oxide production. EcO83‐EVs demonstrate significant potential as a postbiotic alternative to live bacteria, offering a safer option for therapeutic applications. Further research is required to explore their clinical use, particularly in mucosal vaccination and targeted immunotherapy strategies.

## Graphical abstract

This study investigates *Escherichia coli* A0 34/86 (EcO83), a probiotic strain known for its ability to prevent infections and diarrhoea in newborns and reduce allergic airway inflammation in mice. Although live probiotic bacteria are effective, they also harbour certain risks, such as possible infection and gene transfer. Therefore, this research investigates the use of extracellular vesicles (EVs) from EcO83 (EcO83‐EVs) as a safer alternative. The EVs were isolated and characterised in accordance with the MISEV guidelines. Experiments using human nasal epithelial cells, a mouse bone marrow‐derived macrophage cell line and mice showed that EcO83‐EVs promote the expression of immunomodulatory proteins and oxidative stress, and involve activation of the NF‐κB signalling pathway and nitric oxide production. These results suggest that EcO83‐EVs may represent a promising postbiotic therapeutic option with potential applications in mucosal vaccination and immunotherapy. However, further research is required to assess their clinical suitability.

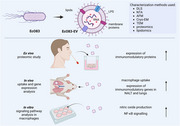

## 1 INTRODUCTION

The strain *Escherichia coli* A0 34/86, serotype O83:K24:H31 (EcO83) is a probiotic bacterium (Colinfant New Born, Dyntec, Czech Republic) marketed for use in newborns and infants at risk of nosocomial infection and diarrhoea (Súkeníková et al., 2023; Wassenaar, 2016). Oral administration is well tolerated and leads to successful and long‐term colonisation of the gut (Lodinová‐Zádníková et al., 2003). It is increasingly evident that the gut microbiota can trigger an immunological response across multiple tissue sites, and their local persistence can activate cells in distant tissue sites (Ngo et al., 2024). In this context, the immunological cross‐talk between the gut and lung was tested in infants of allergic mothers by the oral administration of live EcO83 (Lodinová‐Žádníková et al., 2010). Although EcO83 reduced the development of atopic skin disease in these children, no beneficial effects were shown on allergy‐related lung pathology. Conversely, we have previously shown that live probiotic bacteria, when administered directly into the nasal cavity, induce a strong humoral and cellular response in the lung (Sarate et al., 2021; Sarate et al., 2019; Schabussova et al., 2011).

Mechanistically, we have previously demonstrated that EcO83 signals via Toll‐Like Receptor (TLR) 2 and TLR4 (Schmid et al., 2023), induce pro‐ and anti‐inflammatory cytokines in human and mouse antigen‐presenting cells, such as dendritic cells and macrophages (Schmid et al., 2023; Súkeníková et al., 2017). Using the mouse macrophage cell line RAW264.7, we have shown that genes related to the TLR4 signalling pathway, such as IL‐6, TNF‐α, and IL‐1β, are upregulated by EcO83 (Schmid et al., 2023). When administered intranasally, EcO83 modulates lung cell composition and cytokine production and, importantly, provides protective effects against airway allergy in a TLR4‐dependent manner (Zwicker et al., 2018). Considering the potential risks associated with the use of live bacteria, such as bacterial gene transfer or systemic infections in vulnerable populations (Kothari et al., 2019), strategies utilising non‐viable bacterial‐derived structures, so‐called postbiotics, have been proposed (Schmid et al., 2023). These postbiotics may provide similar benefits to live bacteria while mitigating the risks associated with their use and being easier to control in terms of their therapeutic dosage.

Extracellular vesicles (EVs) are membrane‐enclosed structures that can interact with pattern recognition receptors (PRRs) through their diverse and biologically active cargo (Kuipers et al., 2018). EVs produced by Gram‐negative bacteria contain an array of molecules including proteins, lipids, nucleic acids, peptidoglycan, and lipopolysaccharide (LPS) (Kim et al., 2015). LPS is recognised by TLR4 and induces the production of Th1 cytokines via the nuclear factor‐κΒ (NF‐κΒ) signalling pathway (Aderem & Ulevitch, 2000). In our recent study, we have shown that EcO83 produce EcO83‐EVs which we have isolated by ultracentrifugation (UC) (Schmid et al., 2023). These crude EcO83‐EVs signal via TLR4 and induce the expression of downstream genes in the NF‐κΒ signalling pathway (Schmid et al., 2023). TLR4 agonists have been shown to be efficient mucosal adjuvants that induce robust humoral antigen‐specific responses and stimulate an antigen‐specific Th1 immune response when administered intranasally (Bakkari et al., 2021; Przetak et al., 2003). NF‐κΒ signalling pathway regulates genes associated with cellular stress responses, antioxidant effects, or anti‐apoptotic activities (Zaph et al., 2007). In the airways, the same signalling pathways are responsible for the induction of genes linked to inflammation, which subsequently orchestrate oxidative stress responses and trigger signalling cascades in the lung (Tang et al., 2021). One potent factor induced by the NF‐κΒ signalling pathway is nitric oxide (NO), a versatile molecule influencing a spectrum of physiological processes including immunity, metabolic functions and neurotransmission (Lundberg & Weitzberg, 2022). Macrophages exposed to bacterial products such as LPS produce NO (Nathan & Xie, 1994), which not only exerts a microbicidal effect (Nathan & Hibbs, 1991) but also plays a critical role in shaping the adaptive immune response (Bogdan, 2015).

To decipher the mechanisms of vesicle‐host interaction, we first isolated, purified, and characterised EVs from EcO83 (EcO83‐EVs), according to the Minimal Information for Studies of Extracellular vesicles (MISEV) guidelines (Welsh et al., 2024). We exposed human primary nasal epithelial cells ex vivo to EcO83‐EVs and detected an upregulation of proteins related to oxidative stress and inflammatory responses. To further investigate these interactions, we analysed the dynamics between EcO83‐EVs and nasal‐associated lymphoid tissue (NALT), as well as lung tissue, following the intranasal administration of these EVs in a mouse model in vivo. Our results show that EcO83‐EVs trigger an immune response in NALT and drive neutrophil recruitment to the lung. Furthermore, we demonstrate that EcO83‐EVs are actively internalised by airway macrophages in vivo. In vitro studies revealed that EcO83‐EVs induce NO production by macrophages via the NF‐κΒ signalling pathway.

## 2 MATERIALS AND METHODS

### 2.1 Bacteria

EcO83 was grown in a Brain‐Heart Infusion broth medium (BHI, Sigma Aldrich, USA). The culture was inoculated with a sample from a frozen glycerol stock and incubated at 200 rpm, overnight (ON), at 37°C (Innova 4230 Incubator, Eppendorf New Brunswick, Germany). The colony forming units (CFU) were calculated using a growth curve established in‐house. For cell culture experiments, bacteria were inactivated with 1% paraformaldehyde (PFA; SAV Liquid production, Germany) for 3 h at room temperature (RT), washed twice in phosphate‐buffered saline (PBS; Gibco, Thermo Fisher Scientific, USA), and stored at ‐20°C until further use. Bacterial lysates were prepared from 10 mL of EcO83 culture at an OD of 1.98 (2.8 × 10^8^ CFU/mL). The 10 mL culture was spun down, washed twice with PBS, dissolved in 1 mL lysis buffer (20 mM Tris‐HC, 0.14 M NaCl, 10% glycerol, protease inhibitor tablet cOmplete (Roche, Switzerland)), transferred to a bead beating tube (VK01, Bertin Technologies, France) and homogenized in a Precellys beadbeater (Bertin Technologies) at 6500 rpm for 30 s. The tubes were centrifuged at maximum G force, and the supernatant was collected and stored at ‐20°C until further use.

### 2.2 Isolation, purification, rhodamine labelling, and polymyxin treatment of EcO83‐EVs

**FIGURE 1:**
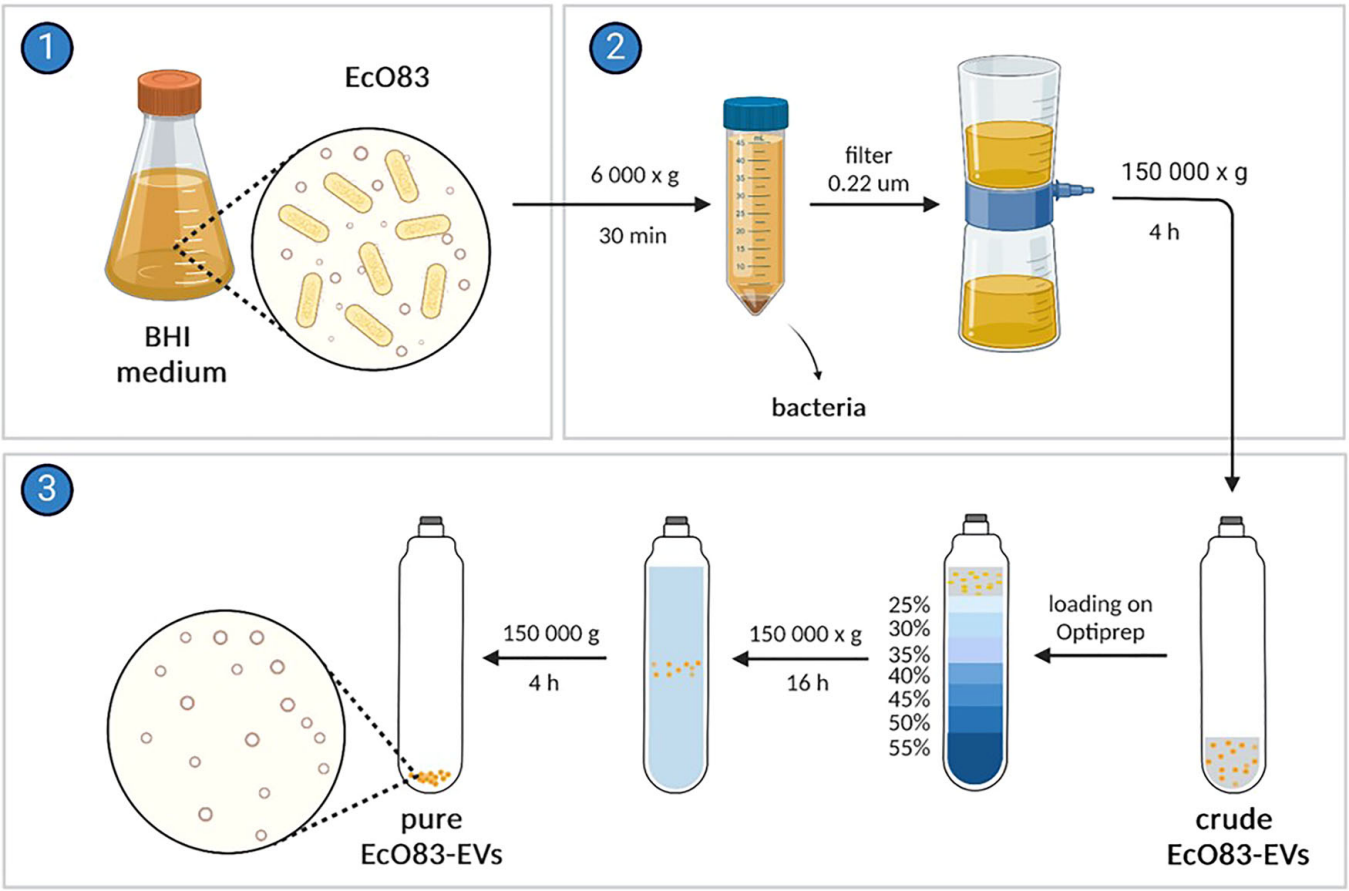
Production and purification of EcO83‐EVs. EcO83 was grown in a BHI medium for 8 h, the bacteria culture were spun down, and the supernatant was filtered through a 0.22 µm pore membrane. The filtrate was then ultracentrifuged and purified on the OptiPrep gradient. Collected fractions were ultracentrifuged, characterized and pooled to obtain pure EcO83‐EVs. Created with BioRender.

EcO83‐EVs were isolated according to Schmid et al. (Schmid et al., 2023) (Figure 1). Briefly, EcO83 was grown in BHI medium with shaking at 200 rpm, ON, at 37°C. The main culture was inoculated at a ratio of 1: 100 from the ON culture and grown for 8 h under the same conditions. The bacteria initially showed logarithmic growth. We stopped the culture when it was in the stationary phase. The bacterial culture was centrifuged at 5000 × *g*, 15 min, at 4°C (Heraeus, Thermo Fisher Scientific). The supernatant was filtered through a 0.22 µm pore filter (Merck Millipore, USA). The crude EcO83‐EVs were obtained by UC (150 000 × *g*, 3 h, at 4°C; Beckman Coulter, USA, 45Ti rotor). The pellet was dissolved in 500 µL sterile 0.9% NaCl (B. Braun, Austria) and stored at 4°C until purified.

The crude EcO83‐EVs were further purified by a density gradient (DG) of 25% to 55% OptiPrep (Iodixanol; Serumwerk, Germany) in ddH_2_O, 1.4 mL/fraction, sample loaded on top) UC at 150,000 × *g*, 16 h, 4°C (Beckman Coulter, Sw41 Ti Swinging Bucket Rotor). Nine fractions of 1.2 mL were taken starting from the top, diluted in 60 mL saline, and UC again at 150,000 × *g*, 3 h, at 4°C (Beckman Coulter, 45Ti rotor). The EcO83‐EVs were resuspended in saline, and the fractions were analysed for particle number, size, protein‐ and LPS content. Fractions containing EcO83‐EVs (F4‐F7) were pooled (Figure S1), characterized, and stored at 4°C until further use. BHI medium without bacteria was subjected to the same procedure to collect mock‐EVs.

For rhodamine labelling, crude EcO83‐EVs were incubated ON with 0.9 mg/mL octadecyl rhodamine B chloride (R18) (Invitrogen, USA) in labelling buffer (50 mM Na_2_CO_3_, 100 mM NaCl, pH 9.2) with shaking at 200 rpm, ON, at 20°C. The next day, EcO83‐EVs were purified using the OptiPrep DG as described above, but with rhodamine‐labelled crude EcO83‐EVs loaded onto the bottom of the gradient. Fraction F6 was collected and characterized. For polymyxin pre‐treatment, Ec083‐EVs were incubated with 20 µg/mL of polymyxin B (Sigma‐Aldrich) for 30 min.

### 2.3 Size, concentration, and zeta potential measurements

The size and particle concentration of the purified, monodisperse EcO83‐EVs and EcO83 were analysed using the Zetasizer Ultra Red Label (Malvern Panalytical, UK). Zetasizer working ranges were of: concentration measurement range of 1 × 10^8^ to 1 × 10^12^ particles/mL and diameter of 0.3 nm ‐ 15 µm. The measurements were carried out without diluting the sample under aseptic conditions in a small‐volume quartz batch cuvette (ZEN2112, Malvern Panalytical). Each measurement was done using General Mode and in triplicate. EcO83‐EV´s zeta potential (ZP) was measured using a disposable folded capillary cell (DTS1070, Malvern Panalytical). The sample was diluted 1: 500 (or 1: 3000 for bacteria) in 25 mM HEPES and measured in triplicate. Measurement quality and data analysis were performed using Malvern ZS Xplorer software (version 3.2.0.84).

Nanoparticle Tracking Analysis (NTA) was used to complement the size measurement of EcO83‐EVs. The analysis was performed with the Nanosight instrument (Malvern Panalytical). The sample was diluted 1: 200 in 0.9% NaCl; 156.6 particles per frame were measured; laser type Blue488; camera type sCMOS; camera level 13; NTA version NTA 3.2 Dev Build 3.2.16.

### 2.4 Cargo analysis

#### 2.4.1 Protein concentration and SDS‐PAGE

Protein concentration was determined using the Bradford protein assay (BioRad, USA) according to the manufacturer's protocol, and absorbance was measured with TECAN Spark 10 M plate reader (Tecan Trading AG, Switzerland). Protein content was re‐calculated for 1 × 10^11^ EcO83‐EVs. The protein profile in EcO83‐EVs fractions was analysed by SDS‐PAGE using NuPAGE 4–12% precast gels (Thermo Fisher Scientific) and stained with SimplyBlue SafeStain (Invitrogen).

#### 2.4.2 Proteomics of EcO83‐EVs

Proteomics analyses of EcO83‐EVs and EcO83 cell lysates were performed as previously described (Schmid et al., 2023). Briefly, 20 µg of protein was dissolved in Laemmli buffer, and separated by SDS‐PAGE. Reduced cysteine residues were blocked with iodoacetamide (Sigma Aldrich, USA) and proteins were digested by trypsin (Sigma Aldrich) in the gel. Peptides were purified on PepClean C18 Spin Columns (Thermo Fisher Scientific) and dried using a Speedvac, miVac Duo Concentrator (Genevac, UK). Peptides were analysed using a nano‐LC (Easy‐nLC 1200, Thermo Fisher Scientific)‐tandem‐MS (Q‐Exactive HF‐X Hybrid Quadrupole Orbitrap, Thermo Fisher Scientific). MaxQuant (v1.5.3.30) (Tyanova et al., 2016) was used to identify (Götz et al., 2008) and quantify proteins against the *E. coli* K12 database (https://www.uniprot.org/). Matching between runs was performed and iBAQ intensities were used for quantification and processed in Perseus v2.0.11 (Tyanova et al., 2016). Proteins were filtered with a permutation‐based false discovery rate (FDR). Volcano plots were generated using GraphPad Prism v10. Bioinformatic analysis was performed using OmicsBox (BioBam, Spain) with GO enrichments determined by Fisher's exact test comparing proteins upregulated in EcO83‐EVs versus EcO83 lysates against a background of all proteins identified during proteomic analysis of both EcO83‐EVs and EcO83 lysates.

The mass spectrometry proteomics data have been deposited to the ProteomeXchange Consortium via the PRIDE (Perez‐Riverol et al., 2022) partner repository with the dataset identifier PXD052178.

### 2.5 Lipopolysaccharide measurement

The endotoxin (LPS) content was analysed using the PyroGene Recombinant Factor C Endpoint Fluorescent Assay (Lonza, Switzerland) according to the manufacturer's instructions. Fluorescence was measured with a TECAN Spark 10 M plate reader. LPS content was re‐calculated for 1 × 10^11^ EcO83‐EVs.

### 2.6 Measurement of total lipids

The quantification of total lipids in EcO83‐EVs was performed according to Visnovitz et al. (Visnovitz et al., 2019). The total lipid extract *E. coli* (ATCC 11303) was used as a standard (Avanti Polar Lipids, USA). The lipid standard was resuspended in chloroform (Sigma Aldrich), evaporated at 60°C and transferred to 25 mM HEPES (Gibco). A standard curve was established: 1 µg ‐ 32 µg lipid per 40 µL sample. Two hundred microliters of concentrated H_2_SO_4_ (Sigma Aldrich) was added to 40 µL of sample/standard, vortexed, and incubated at 90°C for 20 min in open tubes. Samples were cooled, 120 µL of phospho‐vanillin (Sigma Aldrich) was added (0.025% vanillin in 17% phosphoric acid), and vortexed. Two hundred and eighty microliters of each sample was transferred to a 96‐well plate, and the reaction was allowed to develop for 1 h, at 37°C. Absorbance was measured at 540 nm by a TECAN Spark 10 M plate reader. Lipid content was re‐calculated for 1 × 10^11^ EcO83‐EVs.

### 2.7 Lipidomic analysis

Time of Flight—Secondary Ion Mass Spectrometry (ToF‐SIMS) was used to perform a comparative analysis of the surface lipid content of EcO83‐EVs and parent bacteria. ToF‐SIMS 5 instrument (ION‐ToF GmbH, Germany) was used in the so‐called *static mode* with ionized bismuth clusters (Bi_3_^+^) at 30 keV voltage. The positive mass spectrum was recorded in the mass range of up to 900 Da at a current of 1.10 pA (Kamińska et al., 2021). Thirty microliters of duplicates of each sample were applied to a silicon surface (Sigma Aldrich) with a 1 × 1 cm^2^ size. Three surface measurements of 150 × 150 µm^2^ for positive ions (125 × 125 pixels) were performed for each sample. Two 50 × 50 µm^2^ regions of interest (ROI) were selected for each measurement, and 2D emission maps for ions characteristic of phosphocholine fragments (*m/z* 184.2) were examined to eliminate local artefacts. The total ion dose deposed to the surface of interest normalised the data. Each spectrum was calibrated using the positive ion signals: H^+^, H_2_^+^, CH^+^, CH_2_^+^, CH_3_^+^, and C_3_H_2_^+^. SurfaceLab software (version 7.2) and OriginPro (ver. 9.8.0.200, 2020b) were used to analyse the data. The data supporting this study's findings are openly available in the RODBUK database of Jagiellonian University in Krakow at https://doi.org/10.57903/UJ/RLIHE7.

### 2.8 RNA analysis

1.6 × 10^11^ EcO83‐EVs were treated with proteinase and RNAse to digest external RNA using a method adapted from Hu et al. (Hu et al., 2021). In brief, EcO83‐EVs were treated with 100 µg/mL proteinase K (Sigma Aldrich) for 2 h, at 37°C, which was then inactivated for 1 h, at 75°C. Subsequently, 10 U/mL RNase ONE (Promega, USA) was added for 30 min at 37°C. The RNAse was then inactivated with proteinase K, which was subsequently inhibited with the protease inhibitor cOmplete (Roche) for 1 h, at RT. The RNA was extracted with a qEV RNA extraction kit (IZON Science, France) according to the manufacturer's protocol. In parallel to the isolation of RNA from EcO83‐EVs samples, RNA was also isolated from a sample of EcO83. The extracted RNA was analysed with the 2100 Bioanalyzer (Agilent, USA) using the 6000 Nano kit according to the manufacturer's protocol and stored at ‐80°C for further use. The RNA content was re‐calculated for 1 × 10^11^ EcO83‐EVs.

### 2.9 DNA analysis

The DNA was isolated from the samples using the FavorPrep Tissue Genomic DNA Extraction Mini Kit (Favorgen, Taiwan) according to the manufacturer's protocol. In parallel to the isolation of DNA from EcO83‐EVs samples, DNA was also isolated from a sample of PFA‐fixed EcO83. Prior to DNA isolation, EcO83‐EVs were treated with 100 µg/mL proteinase K (Sigma Aldrich) for 2 h, at 37°C to digest all proteins on the surface, followed by heat inactivation of the enzyme for 1 h, at 75°C. DNAse (DNA‐free kit, Thermo Fisher Scientific) was then added to digest DNA on the surface of the EVs and incubated for 30 min, at 37°C. The DNAse was inactivated using the DNAse inactivation reagent provided in the kit according to the manufacturer's instructions. DNA was isolated from two samples of 1.2 × 10^11^ EcO83‐EVs: treated with DNase and proteinase K and treated the same way without the addition of DNase and proteinase K. The extracted DNA was loaded onto a 2% agarose gel with a DNA stain Midori Green (Nippon Genetics, Germany). The samples were mixed with 6X Gel Loading Dye (New England Biolabs, USA). The gel was run at 150 V for 45 min and photographed with a UV camera (Peqlab, Germany).

### 2.10 Transmission Electron Microscopy (TEM)

EcO83‐EVs were negatively stained to visualize them at the electron microscopic level, as previously published (Schmid et al., 2023). Briefly, 5 µL of EcO83‐EVs were allowed to adhere to formvar‐ and carbon‐coated grids for 20 s. To contrast the samples, 5–10 µL of 1% aqueous uranyl acetate was added and simultaneous absorption of the liquid with a filter paper was performed twice on each grid (single drop method). The air‐dried grids were immediately imaged in an FEI Tecnai20 electron microscope (FEI, Netherlands) using a 4K Eagle‐CCD camera. Images were processed with Adobe Photoshop.

### 2.11 Cryogenic Electron Microscopy (cryo‐EM)

Cryo‐EM samples of EcO83‐EVs were prepared on Quantifoil EM grids (Electron Microscopy Sciences, USA), on which 10 nm protein A colloidal gold particles (Au–NP) were pre‐adsorbed (Aurion, Netherlands). The Au–NP adsorbed grids were glow‐discharged (30 s, 40 mA) in a Quorum GLOQUBE Plus system. An aliquot (2 µL) of the aqueous solution of the sample was applied to both sides of the EM grids and subsequently blotted for 2 s at blot force ‐7 and plunge‐frozen into the pre‐cooled liquid ethane with a Vitrobot Mark IV (FEI, USA). Cryo‐electron micrographs of the vitrified samples were collected with a JEOL JEM‐2100PLUS transmission electron microscope (JEOL, Japan) equipped with a TemCam‐XF416(ES) (TVIPS, Germany) camera, using an Elsa cryo‐transfer holder model 698 (Gatan) at accelerating voltage of 200 kV. Grid mapping and image acquisition were performed using SerialEM software (Mastronarde, 2005) at a nominal magnification of 80x and 2500x, respectively. High‐magnification images were recorded at 60,000 x nominal magnification (0.194 nm pixel size) with a defocus value of −2.1 µm. To minimize radiation damage during image acquisition, the low‐dose mode in the SerialEM software was used and the electron dose was kept below 100 e^–^/Å^2^.

### 2.12 Atomic Force Microscopy (AFM)

AFM micrographs of EcO83‐EVs were recorded following a procedure described in more detail elsewhere (Ridolfi et al., 2023). Images were acquired in PeakForce mode on a Multimode 8 microscope (Bruker, USA) equipped with Scanasyst Fluid+ probes (Bruker) a Nanoscope V controller, a type JV piezoelectric scanner and a sealed fluid cell. Briefly, EVs were deposited on poly‐L‐lysine functionalized glass coverslips and left to adsorb for 30 min at 4°C, then inserted in the fluid cell without further rinsing. Sample dilution was adjusted in successive depositions to maximize the number of isolated objects. Quantitative morphometry was performed via Gwyddion 2.61 (Nečas & Klapetek, 2012) and custom Python scripts. Two parameters were calculated for each individual object deposited on the surface: the original diameter it had in solution (D) and the contact angle it displayed once adsorbed to the surface (CA). The latter was previously demonstrated to be directly proportional to the mechanical stiffness of intact vesicles (Ridolfi et al., 2023; Ridolfi et al., 2020).

### 2.13 *Ex vivo* treatment of human airway cells with EcO83‐EVs and PFA‐fixed EcO83

#### 2.13.1 Human Nasal Epithelial Cell (NEC) culture

Nasal brush biopsies, NEC passaging, and differentiation at the air‐liquid (ALI) interface were performed according to our previously published protocol (Martens et al., 2019). A total of six study participants were recruited at the Division of Neonatology of the Medical University of Vienna, including three full‐term healthy newborns (gestational age between 37–40 weeks) and three healthy adult controls. The exclusion criteria included the presence of maternal autoimmune diseases, immunological diseases, genetic disorders, and congenital malformations at the time of recruitment. Only healthy, non‐smoking and non‐allergic adults without respiratory infection for 4 weeks before brushing were included. Before stimulation, the apical surface of the ALI was washed with pre‐warmed PBS to remove excess mucus. The cells were stimulated at the apical site with EcO83‐EVs (100 ng/mL according to protein content) or PBS for 24 h, at 32°C in a humidified incubator. After stimulation, the cells were detached using Accutase (Thermo Fisher Scientific) and centrifuged at 400 × *g*, 5 min, at 4°C.

### 2.14 Protein extraction, in‐solution digestion, and TMT‐10plex labelling

Samples were prepared and analysed by LC‐MS/MS as described previously following TMT‐10plex isobaric labelling and off‐line high pH fractionation [Wisgrill et al., accepted in *Allergy*, 2024].

The mass spectrometry proteomics data have been deposited to the ProteomeXchange Consortium via the PRIDE (Perez‐Riverol et al., 2022) partner repository with the dataset identifier PXD052553.

### 2.15 Proteomics data processing

The raw files were imported to Proteome Discoverer v2.4 (Thermo Fisher Scientific) and analysed using the SwissProt protein database with Mascot v 2.5.1 (MatrixScience Ltd., UK) search engine.

Statistical analysis was performed using R Statistical Software (v4.3.2; R Core Team (2023), https://www.r‐project.org). R code is accessible at https://github.com/wisgrilllab/Razim‐et‐al‐TMT‐proteomics. SL normalization, TMM normalization, IRS normalization, and log‐transformation were conducted using the edgeR package (Robinson et al., 2010). The differential expression of proteins was analysed using limma (Ritchie et al., 2015), wherein adjustment for paired samples was implemented within the design matrix. Volcano plots were created using the EnhancedVolcano package (EnhancedVolcano: publication‐ready volcano plots with enhanced colouring & labeling, n.d.). Protein‐protein interactions of the differentially expressed proteins were analysed using the STRING database (Szklarczyk et al., 2023).

### 2.16 In vivo studies

#### 2.16.1 Animals used in the study

Wild type (WT) BALB/c specific pathogen‐free mice (female, 6–8 weeks old) were purchased from Charles River (Germany). The animals were kept in conventional housing at the Institute for Pathophysiology and Allergy Research of the Medical University of Vienna with free access to food and water and with a 12 h light‐dark cycle.

#### 2.16.2 Intranasal treatment of mice with EcO83‐EVs

2.5 × 10^10^ EcO83‐EVs (rhodamine‐labelled or unlabelled) in 40 µL 0.9% NaCl were administered intranasally to mice anesthetized with 5% isoflurane (Animalcare, UK). Mice were then euthanized after 0.5 h or 2 h and the nose‐associated lymphoid tissue (NALT) and lungs were removed. Mice treated with 0.9% NaCl were used as a sham control.

### 2.17 FACS analysis of EV uptake and characterization of immune cells

For the uptake test, lungs were removed from mice under terminal anaesthesia and processed as previously described (Schmid et al., 2023). Lung lobes were minced by gentleMACS Octo Dissociator (Miltenyi Biotec, Germany) and incubated in RPMI‐1640 (Gibco) with 0.05 mg/mL Liberase TL (Roche) and 0.5 mg/mL DNAse (Sigma Aldrich) for 30 min, at 37°C. The digested lung tissue was forced through a 0.70 µm cell strainer, and red blood cells were lysed using ACK Lysing Buffer (Lonza) for 3 min. After lysis, cells were resuspended in complete RPMI (10% FCS, Gibco; 2 mM mercaptoethanol, 2 mM L‐glutamine, and 100 µg/mL gentamicin; Sigma Aldrich). Cell viability and concentration were measured by trypan blue dye‐exclusion using a Neubauer Chamber (Carl Roth, Germany). Next, the lung cells were washed twice with cold PBS. Then 1 × 10^6^ cells/well from each sample were used for the staining and subsequent FACS analysis. Fc receptors were blocked by the addition of anti‐CD16/CD32 (eBioscience, USA), and dead cells were labelled with Fixable Viability Dye eFluor 506 for 20 min, at 4°C in a total volume of 50 µL PBS. Cells were then stained with mAbs for surface markers for 30 min, at 4°C in the dark in 100 µL of PBS, washed, and resuspended in PBS. Cells were analysed using BD FACSCanto II cytometer (BD Biosciences, USA), and data were processed using FlowJo software (BD Biosciences; software version 10.8.1.). Cells from the lungs of control mice were used as natural FMO (fluorescence minus one) for PE+ signal (signal from labelled EVs taken up by the cells). The mAbs used were combined in two panels (all from eBioscience). The lymphoid panel included: TCRβ‐FITC, TCRγδ‐PE‐Cy7, CD19‐APC, CD4‐APC‐Cy7, CD8a‐Pacific Blue. The myeloid panel included: Ly6G (Gr‐1)‐FITC, CD11b‐PE‐Cy5.5, MHC‐II‐PE‐Cy7, Siglec‐F‐AF 647, Ly6C‐APC‐eFluor780, CD103‐eFluor450.

For the study of lung cell populations after intranasal treatment, lungs were minced using scissors and further processed as described above. They were seeded in 96‐well U‐bottom plates (1 × 10^6^ cells/well). Fc receptors were blocked as described above and dead cells were stained using the 7‐AAD viability dye (eBioscience). Cells were analysed on a Northern Lights Spectral Flow Cytometer (Cytek, USA) and data were processed using SpectroFlo Software (Cytek). The antibody panel included (all eBioscience unless stated otherwise): CD4‐FITC, CD8‐eFluor‐450, CD19‐eFluor 405, TCRγδ‐SuperBright 780, CD3‐SuperBright 702, CD11b‐APC‐Cy7, CD11c‐PE, SiglecF‐AlexaFluor 647 (BD Biosciences), Ly6C‐APC, Ly6G‐PE‐Cy5, CD103‐PE‐Cy7, CD49b‐PerCP‐Cy5.5 (BioLegend, USA), and MHC‐II‐PerCP‐eFluor710.

### 2.18 qPCR analysis

NALT and a piece of the right lung lobe were used for RNA isolation with the RNeasy Mini Kit (Qiagen, Germany) according to the manufacturer's instructions. Concentrations of total RNA were measured using the Nanodrop 1000 (Thermo Fisher Scientific) and 1 µg of RNA was used for reverse transcription. The cDNA was obtained by the iSCRIPT cDNA Synthesis Kit (Bio‐Rad) using the T100 thermal cycler (Bio‐Rad) following the manufacturer's protocol. The qPCR was performed in duplicates using the SsoFast qPCR supermix (Bio‐Rad), according to the manufacturer's protocol, and primers listed in Table 1 using the CFX Duet RT‐PCR system (Bio‐Rad). A cycle threshold (Ct) value was determined for each sample, and the average of duplicates was calculated. Primer sequences were obtained from PrimerBank (Spandidos et al., 2010) or designed in‐house. The 2^−ΔΔCt^ method was used to calculate the relative expression of each target gene. Gene β‐actin (*Actb*) was used as a reference to normalize the gene expression data.

**TABLE 1:** List of used primers for the q‐PCR.

Name of the primer	PrimerBank ID	Primer sequence 5′ to 3′
mNF‐κΒ 2 fwd	9506921a1	GGCCGGAAGACCTATCCTACT
mNF‐κΒ 2 rev	9506921a1	CTACAGACACAGCGCACACTs
miNOS fwd	6754872a1	GTTCTCAGCCCAACAATACAAGA
miNOS rev	6754872a1	GTGGACGGGTCGATGTCAC
mIL‐6 fwd		CACTTCACAAGTCGG
mIL‐6 rev		CTGCAAGTGCATCATCGTTGT
mIL‐10 fwd		CAGAGCCACATGCTC
mIL‐10 rev		TCATTTCCGATAAGG
mBCL‐3 fwd	15809014a1	CCGGAGGCCCTTTACTACCA
mBCL‐3 rev	15809014a1	GGAGTAGGGGTGAGTAGGCAG
mSOD2 fwd	31980762a1	CAGACCTGCCTTACGACTATGG
mSOD2 rev	31980762a1	CTCGGTGGCGTTGAGATTGTT
mCCL19 fwd	6755422a1	GGGGTGCTAATGATGCGGAA
mCCL19 rev	6755422a1	CCTTAGTGTGGTGAACACAACA
mβ‐act fwd		GCTCTTTTCCAGCCT
mβ‐act rev		CTTCTGCATCCTGTC

### 2.19 In vitro studies

#### 2.19.1 Cells and culture condition

Mouse bone marrow‐derived macrophage (BMDM) cell line, TLR2‐ (BMDM/TLR2‐), TLR4‐ (BMDM/TLR4‐), TLR7‐ (BMDM/TLR7‐), and TLR9‐ (BMDM/TLR9‐) deficient BMDM cell lines (Bei Resources, NIAID, NIH, USA) were maintained in complete culture medium (CCM) consisting of Dulbecco's modified Eagle's medium (DMEM, Gibco), 10% fetal bovine serum (FBS, Eurx, Poland), penicillin/streptomycin (BioWest, France) and 3% L‐glutamine (Gibco). Cells were grown under standard conditions in a humidified incubator at 37°C in an atmosphere of 5% CO_2_/95% air. Adherent cells from confluent cultures were detached by trypsin + ethylenediaminetetraacetic acid (EDTA; Sigma Aldrich), centrifuged at 150 × *g*, for 10 min, at RT and suspended in CCM.

#### 2.19.2 *MTT* colorimetric assay

Cell viability was determined using an 3‐(4,5‐dimethylthiazol‐2‐yl)‐2‐5‐diphenyltetrazolium bromide (MTT) colorimetric assay (Mosmann, 1983). BMDM cells were suspended in CCM, seeded onto a 96‐well plate (1 × 10^4^ cells/mL), and incubated at 37°C in the atmosphere of 5% CO_2_/95% air. The next day, the medium was replaced with fresh medium, and the cells were stimulated with EcO83‐EVs (1.6 × 10^9^ and 1.6 × 10^10^ EVs/mL) or PFA‐fixed EcO83 (1 × 10^6^/mL and 1 × 10^7^/mL). After 24 h of incubation, the cells were incubated with 5 mg/mL MTT reagent (Sigma Aldrich) for 4 h, at 37°C. Finally, 100 µL of dimethyl sulfoxide (Sigma Aldrich) was added to the plate to dissolve the formazan crystals that had formed and accumulated in the cells. Absorbance was measured using an EnSpire 2300 microplate reader (Perkin Elmer, USA) at 570 nm. Viability was expressed as a percentage of live cells compared to untreated control cells (100%).

#### 2.19.3 Nitric oxide and cytokine generation

The nitrite/nitrate formation test was performed as previously published (Zabłocka et al., 2024). BMDM or BMDM/TLR‐ cells (1 × 10^6^ cells/mL) were seeded on a 12‐well plate and cultured in CCM for 24 h. CCM was then exchanged and EcO83‐EVs (1.6 × 10^9^ and 1.6 × 10^10^ EVs/mL) or inactivated bacteria EcO83 (1 × 10^6^/mL and 1 × 10^7^/mL) were added. 1 µg/mL LPS of *E. coli* serotype O55B5 (Sigma Aldrich) served as a positive control, while untreated BMDM cells were used as a negative control. Nitric oxide (NO) is synthesized by inducible nitric oxide synthase (iNOS); therefore, the selective iNOS inhibitor S‐methylisothiourea (S‐MIU) (10 µM) (Sigma Aldrich) was used to test the specificity of NO production. To determine the impact of extracellular signal‐regulated kinases 1/2 (ERK1/2) and c‐Jun N‐terminal kinases (JNK) activation on the regulation of NO production, BMDM cells were first pre‐incubated with selective kinase inhibitors for 2 h: U0126 (Cell Signaling Technology, Netherlands; 20 µM; for ERK1/2), and SP600125 (Med Chem Express, USA; 25 µM; for JNK) and then stimulated with EcO83‐EVs (1.6 × 10^9^ and 1.6 × 10^10^ EVs/mL) or inactivated bacteria EcO83 (1 × 10^6^/mL and 1 × 10^7^/mL). After 24 h incubation at 37°C, the cell culture supernatants were centrifuged at 6000 × *g*, for 5 min, at RT, and the cell‐free supernatant was assayed for determination of NO concentration and cytokines: TNF‐α, IL‐6, and IL‐10.

#### 2.19.4 Nitric oxide and cytokine determination

NO production was measured by determining the nitrite concentration in the supernatants of cultured BMDM cells using the colorimetric Griess method (Guevara et al., 1998). Briefly, 100 µL samples of cell culture supernatants were incubated with an adequate volume of Griess reagent (0.1% N‐(1‐naphthyl)‐ethylenediamine (Serva Feinbiochemica, Germany) and 1% sulphanilamide (Sigma Aldrich) in 5% phosphoric acid (Avantor, Poland)). After 10 min incubation at RT, the absorbance was measured at 550 nm. The concentration of nitrite was determined by comparison with the NaNO_2_ (Merck) standard curve (0–75 µM).

Cytokine levels (TNF‐α, IL‐6, and IL‐10) in cell culture supernatants were determined using mouse DuoSets (R&D system, USA) according to the manufacturer's instructions.

### 2.20 Western blot

BMDM cells (1 × 10^6^ cells/mL) were seeded on 6‐well culture plates and incubated with EcO83‐EVs (1.6 × 10^9^ and 1.6 × 10^10^ EVs/mL) or inactivated bacteria EcO83 (1 × 10^6^/mL and 1 × 10^7^/mL) for 15–120 min to activate ERK1/2, JNK, and transcription factor NF‐κΒ, and 24 h to stimulate iNOS expression. After stimulation, cells were lysed with RIPA buffer (Sigma Aldrich) supplemented with a protease inhibitor cocktail (cOmplete, Sigma Aldrich), and 1 mM NaF (Sigma Aldrich) on ice for 30 min. The lysates were centrifuged at 14,000 × *g*, for 10 min, at 4°C. The protein content was determined by the bicinchoninic acid (BCA) method (Thermo Fisher Scientific) according to the manufacturer's protocol. Next, the 30 µg protein samples were separated on a 4%–12% sodium dodecyl sulphate (SDS)—polyacrylamide gel (TGX FastCast Acrylamide solutions (Bio‐Rad)), and then transferred to a 0.22 µm nitrocellulose membrane (Bio‐Rad). The membrane was blocked in Tris—HCl buffer, pH 7.0, 5% Tween 20 (TBST) and 5% non‐fat dried milk for 1 h, at RT, then incubated ON, at 4°C with primary antibodies diluted in TBST with 5% BSA: anti‐iNOS (1: 1000) (Santa Cruz Biotechnology, USA), anti‐ERK1/2 (1: 1000), anti‐phospho ERK1/2 (1: 1000), anti‐ JNK (1: 1000), anti‐phospho JNK (1: 1000), anti‐NF‐κΒ (1: 1000), anti‐phospho NF‐κB p65 (1: 1000), and anti‐β‐actin (1: 1000) (Cell Signalling Technology, USA). Finally, the membrane was incubated with secondary anti‐rabbit IgG antibodies conjugated with alkaline phosphatase (Cell Signaling Technology) (1: 10,000 in TBST with 5% BSA) for 1 h at RT. Immunocomplexes were visualized using an NBT/BCIP (Roth) substrate and analysed by densitometry using Molecular Imager ChemiDoc MP Imaging System (Bio‐Rad).

### 2.21 Statistical analysis

Unless otherwise noted, statistical analysis was performed using the GraphPad Prism 9.5.1. Software. Comparisons between groups were based on one sample *t*‐test, ordinary one‐way ANOVA tests or Dunnett's comparisons test with a single pooled variance. The value of * *p* ≤ 0.05 was considered statistically significant.

### 2.22 EV‐TRACK

We have submitted all relevant data of our experiments to the EV‐TRACK knowledgebase (EV‐TRACK ID: EV240021) (Van Deun et al., 2017). The EV‐METRIC for this manuscript is 86%.

## 3 RESULTS

### 3.1 EcO83‐EVs purified by density gradient ultracentrifugation contain proteins, lipids, LPS, and RNA

Crude EcO83‐EVs were purified by DG‐UC in sterile, endotoxin‐free OptiPrep to remove bacterial debris and medium residues (Figure 1). We collected nine fractions and analysed them for particle concentration and LPS and protein content (Figure S1a‐b). We pooled fractions with the highest concentration of particles (F4‐F7). The density of these fractions ranged from 1.190 to 1.267 g/mL (between 35% and 50% OptiPrep). We plated an aliquot of the pooled EcO83‐EVs fractions on BHI agar plates to confirm the absence of contamination by live bacteria (Figure S1c).

**FIGURE 2:**
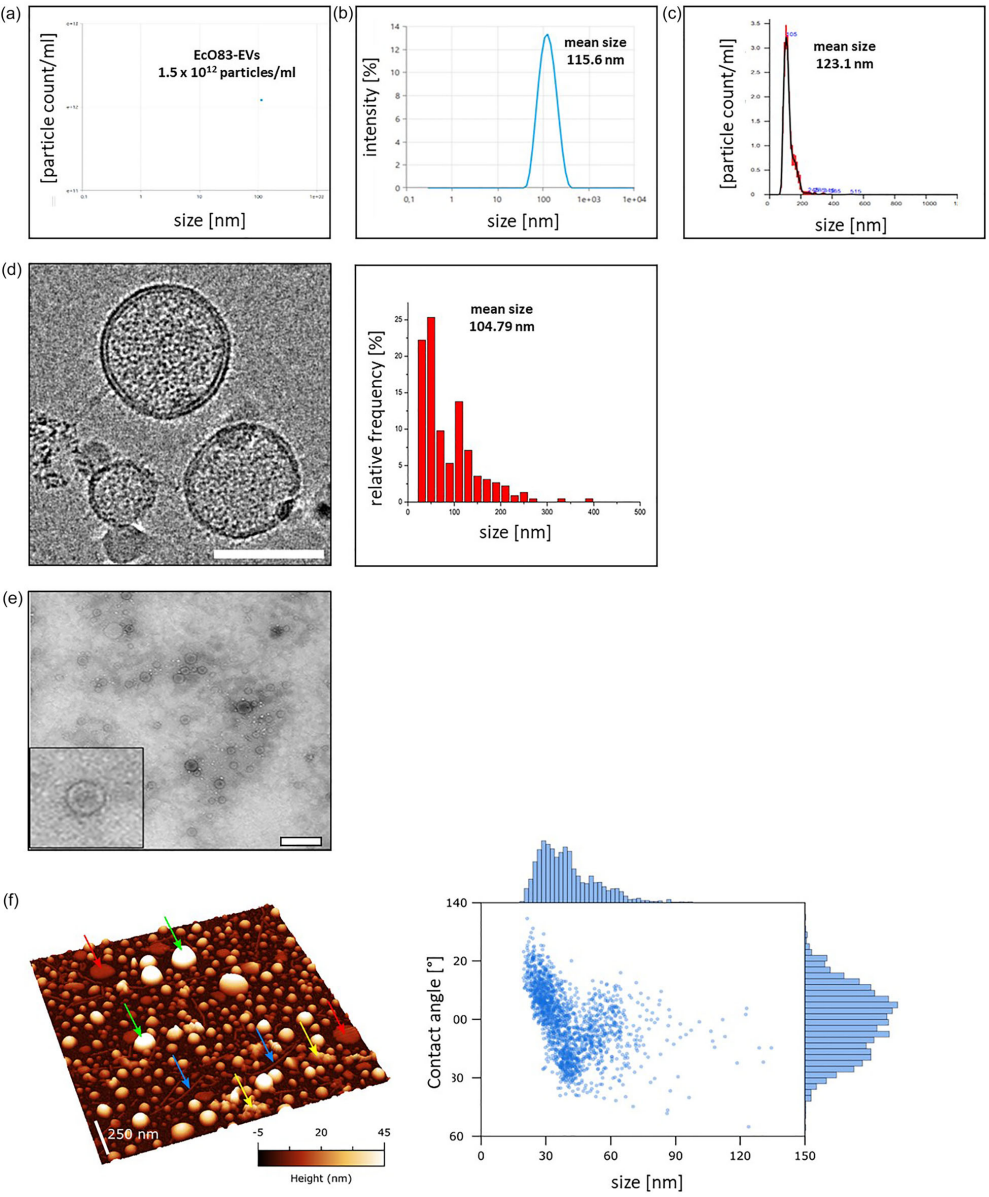
Characterization of EcO83‐EVs. (a) Zetasizer analysis depicting the mean particle concentration of EcO83‐EVs (*n* = 3). (b) Zetasizer particle size measurement of EcO83‐EVs (*n* = 3). (c) Nanoparticle Tracking Analysis of EcO83‐EVs. (d) Cryo‐EM visualization and quantification of EcO83‐EVs (scale bar = 50 nm). (e) TEM image of EcO83‐EVs (scale bar = 200 nm). (f) Left: representative AFM image of EcO83‐EVs (scale bar = 500 nm); green arrows—EVs, blue arrows—fibrillary structures, yellow arrows—amorphous structures, red arrows—deflated EVs. Right: contact angle versus diameter plot of 1760 individual particles found in EcO83‐EVs samples.

From a 2 L EcO83 culture containing 2.67 × 10^12^ bacterial cells, we isolated approximately 0.7 × 10^12^ EcO83‐EVs (in the total volume of 470 µL) (Figure 2a). This equates to isolating roughly 0.26 EVs from each EcO83 bacterial cell. However, the number of vesicles obtained after multiple purification steps serves only as an approximate estimate of the actual vesicle production by bacteria, due to significant vesicle loss during the purification process. We employed multiple analytical methods to measure the size of EVs. We observed that the measurements varied depending on the method used. Using the DLS‐based Zetasizer, the mean diameter of the EcO83‐EVs was found to be 115.6 nm (Figure 2b). Measurements using NTA provided a slightly larger mean diameter of 123.1 nm (Figure 2c), while cryo‐EM yielded an average size of 104.79 nm (Figure 2d). Cryo–EM identified a subpopulation of EVs between 40 and 60 nm in diameter that were not detectable with either NTA or Zetasizer which resulted in the lower mean size of EcO83‐EVs.

We used Cryo‐EM, TEM, and AFM to visualise the EVs (Figure 2d‐f). These analyses confirmed that EcO83‐EVs are intact membrane‐enclosed nanoparticles which assume a largely spherical shape in solution. In particular, cryo‐EM analysis confirmed the presence of a lipid bilayer in EcO83‐EVs (Figure 2d). AFM micrographs allowed for the identification of distinct types of structures (Figure 2f and S2). Sixty percent of the total deposited volume was recognized as globular objects exhibiting an average contact angle of about 100° (Figure S2a‐d), a recurring value in prior measurements of intact EVs (Ridolfi et al., 2020). Ninety‐nine percent of EVs had diameters less than 150 nm, with a typical log‐normal size distribution. Four per cent of the deposited volume consisted of approximately 11 nm thick circular patches that might be EVs damaged upon substrate adhesion (Figure S2e‐g). Another four percent of the deposited volume consisted of long fibrils with a diameter of around 7 nm (Figure S2h‐j). The measured diameter of these fibrillary objects is compatible with the reported dimensions of *E. coli* fimbriae (Hahn et al., 2002), suggesting that these fragments were co‐isolated with EcO83‐EVs. Three percent consisted of marginal amounts of debris composed of particles too small to be assigned any tentative identity (Figure S2a). Around twenty nine percent of the total deposited volume consisted of amorphous aggregates (Figure S2a). Interestingly, a commercial sample of purified LPS deposited under the same conditions exclusively shows very similar aggregates (Figure S2k), suggesting that the aggregates found in EcO83‐EVs fractions might be constituted by LPS.

We found that 1 × 10^11^ EcO83‐EVs contain 67.17 µg of proteins, 69.79 µg of lipids, 1007 µg of LPS, and 16.1 ng of RNA. The vesicular RNA consists predominantly of small RNA fragments of approximately 100 nucleotides in length (Figure S3), indicating the presence of small non‐coding RNA (Lécrivain & Beckmann, 2020). Furthermore, most of the RNA in the EcO83‐EVs appears to be protected from enzymatic degradation, as neither proteinase nor RNase treatment significantly altered the quantity of isolated RNA, as confirmed with Bioanalyzer (Figure S3). In contrast, ribosomal RNA accounts for the majority of the RNA content in the parent bacterium. DNA was detected only in the parent bacteria and not in EcO83‐EVs (Figure S3b).

Since the BHI medium is derived from the infusion of boiled bovine or porcine heart and brain, it could contain eukaryotic EVs. Therefore, we treated the BHI medium in the same way as the supernatant of the EcO83 culture and performed DG‐UC. We pooled fractions F4‐F7 (mock‐EVs) and performed the same analyses as above for the EVs (Figure S4a‐d). Although the mock‐EVs samples contained particles, their concentration was 100‐fold lower compared to the EcO83‐EVs. It is noteworthy that the amount of proteins in this sample was below the detection limit (Figure S4c).

### 3.2 Enrichment of membrane proteins in EcO83‐EVs with conserved lipidomic profile compared to parental bacteria

**FIGURE 3:**
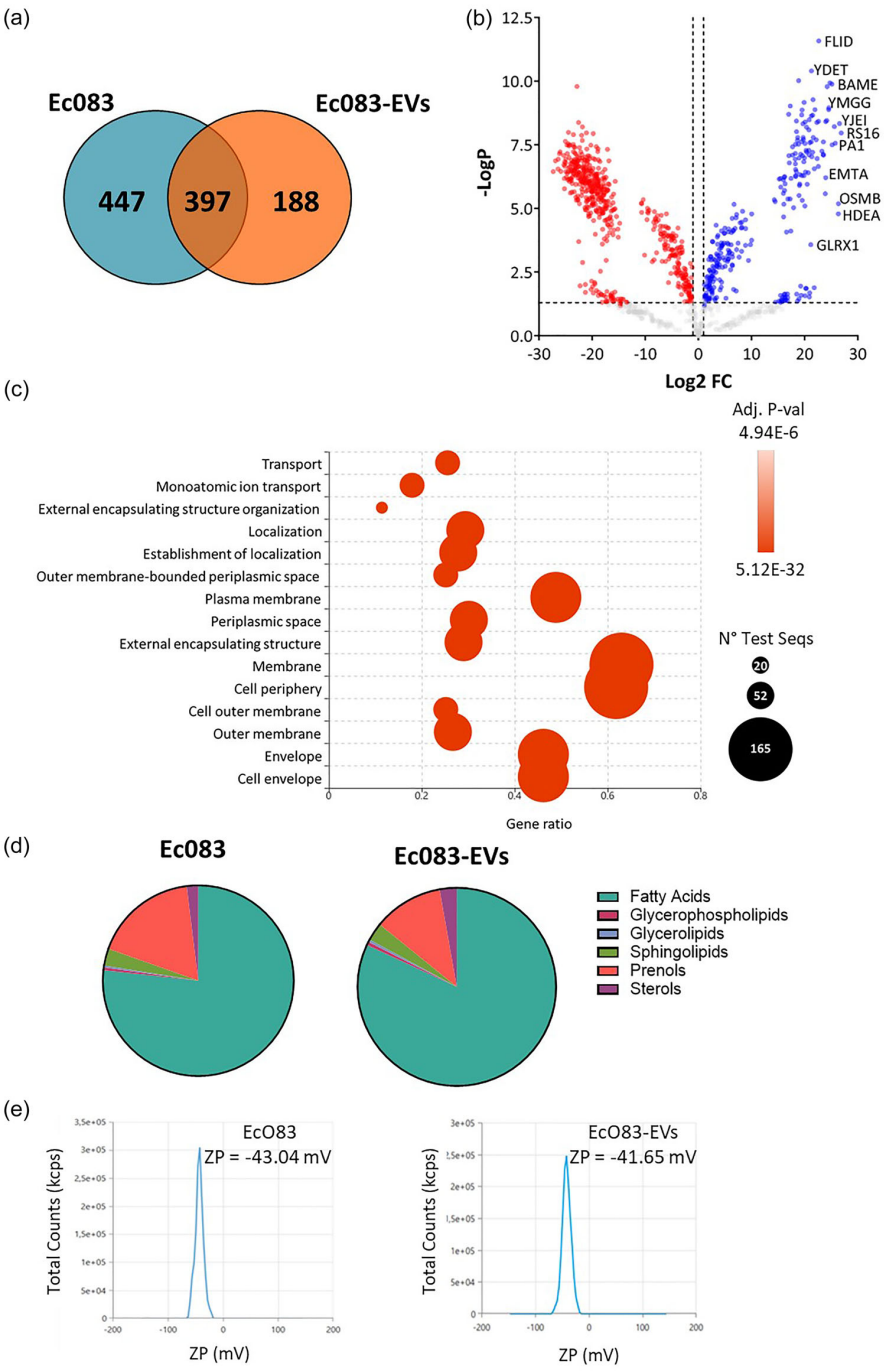
Proteomic and lipidomic analysis of EcO83‐EVs. (a) Venn diagram of proteins identified in EcO83‐EVs and EcO83 bacterial lysate. (b) Volcano plot of proteins upregulated (blue) and downregulated (red) when EcO83‐EVs were compared to bacterial lysate. (c) Bubble plot showing results of Fishers’ test comparing proteins upregulated in EcO83‐EVs against all identified proteins. (d) Lipidomic analysis of the EcO83 and EcO83‐EVs. (e) Zeta potential measurements of EcO83 and EcO83‐EVs.

We performed a comparative proteomic analysis of purified EcO83‐EVs and their parent bacteria. A total of 585 proteins were identified in EcO83‐EVs, with 188 proteins significantly enriched in EcO83‐EVs compared to their parent strain EcO83 (Figure 3a). The particularly enriched proteins include FliD (Flagellar hook‐associated protein 2), YdeT (Fimbrial usher domain‐containing protein), BamE (Outer membrane protein assembly factor), YmgG (UPF0757 protein), YjeL (Uncharacterised protein), RS16 (Small ribosomal subunit protein bS16), PA1 (Phospholipase A1), EmtA (Endo‐type membrane‐bound lytic murein transglycosylase A), OsmB (Osmotically‐inducible lipoprotein B), and HdeA (Acid stress chaperone) (Figure 3b). Further comparative proteomic analyses revealed an enrichment of proteins associated with the membrane, cell periphery, cell wall and membrane organisation (e.g. BamC, LolB, FtsX, or BepA) as well as transporters and transporter components (e.g. FecA, FepA, FecB, FecC, MalM, or MalE) in EVs compared to the parent bacteria (Figure 3c). In contrast to the EcO83‐EVs, no proteins were detected in the mock‐EVs purified by DG‐UC (Figure S4C).

In addition, we analysed the protein composition in EVs before and after DG‐UC by a comparative proteomic analysis (Table S1). 193 proteins were significantly enriched in DG‐UC‐purified than in the crude EcO83‐EVs. A significant number of these enriched proteins are associated with fimbriae, including FimH, FimD, FimA, and FimC. The purified EcO83‐EVs were low in flagellar proteins such as FliC, FlgE, FlgG, and FliK (Table S1). In summary, the purification process reduced the flagellar proteins while it enriched the content of fimbrial proteins in EcO83‐EVs.

Lipidome profiling was performed using ToF‐SIMS, a semi‐destructive, unlabelled, and non‐extractive method enabling the comparison of surface lipids in EcO83‐EVs and their parent bacteria (Marzec et al., 2022). The results revealed that the composition of the main lipid groups in EcO83‐EVs is highly similar to those of the parent bacteria (Figure 3d), indicating that the EVs originate from the outer membrane of the EcO83 bacteria.

To further evaluate and compare the surface properties of EcO83‐EVs and the parent bacteria, we measured their ZP in a 25 mM HEPES buffer. The ZP of EcO83‐EVs was ‐41.65 mV (SD ± 2.932 mV; Quality Factor = 3.64) and that of the parent bacteria was ‐43.04 mV (SD ± 2.295 mV; Quality Factor = 3.927) (Figure 3e), indicating that the surface charge characteristics of the EcO83‐EVs and the parent bacteria are comparable.

### 3.3 EcO83‐EVs induce the expression of proteins associated with inflammation and stress signalling in human nasal epithelial cells

**FIGURE 4:**
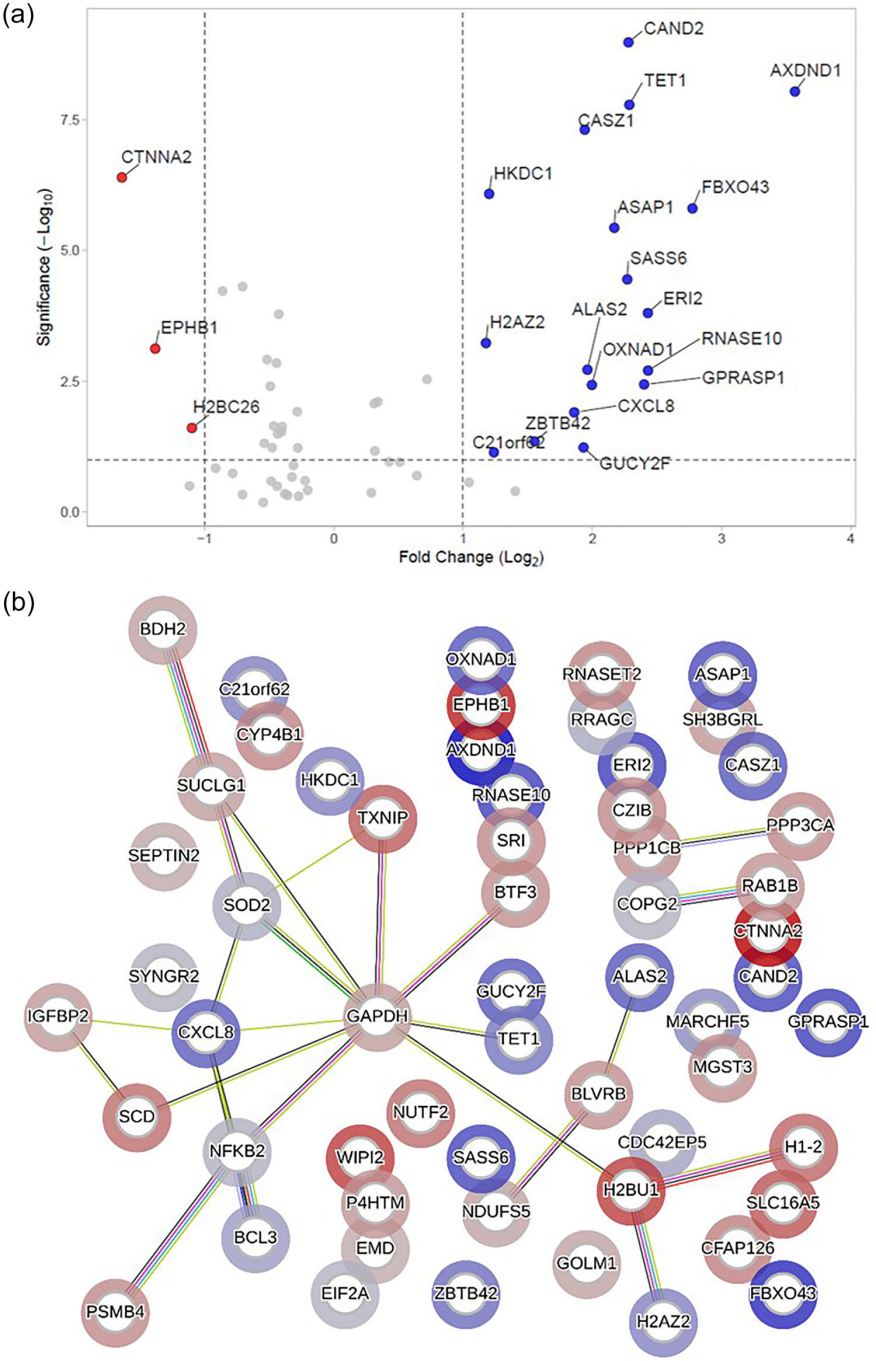
Proteomics of human nasal epithelial cells treated EcO83‐EVs ex vivo. (a) Volcano plot differentially expressed proteins from adult air‐liquid interface nasal epithelial cell cultures after 24 h, at 32°C incubation with 100 ng/mL EcO83‐EVs (according to protein content) compared to PBS‐treated cells. (b) STRING analysis of these differentially expressed proteins. Proteins that are upregulated are circled in blue, and downregulated proteins are shown in red.

To test the interaction of EVs with the host mucosal surfaces, nasal epithelial cells from healthy adult human subjects were differentiated at the air‐liquid interface culture and treated with EcO83‐EVs. Compared to PBS‐treated controls, differential protein expression analysis revealed that 21 proteins were significantly altered in EcO83‐EV‐treated samples (18 were upregulated and 3 were downregulated; *p*‐value ≤0.05) (Figure 4a). According to the Uniprot database, the most upregulated proteins are involved in processes such as protein ubiquitination (CAND2, FBXO43), stress response (HSP90AA4P, ALAS2, OXNAD1), inflammation (CXCL8), and transcription regulation (CASZ1, H2AZ2, ZBTB42). Additionally, proteins such as BCL3 (B‐cell lymphoma 3‐encoded protein), NF‐κΒ2 (Nuclear factor NF‐kappa‐B p100 subunit), and SOD2 (Superoxide dismutase 2), which are linked to IL‐8 (CXCL8) expression, were upregulated, albeit to a lesser extent (Figure 4a). STRING analysis showed the possible functional interactions between upregulated proteins (Figure 4b).

Given the clinical use of EcO83 for prevention of nosocomial infections in newborns, we additionally performed a proteomic analysis of EcO83‐EVs‐treated ALI cultures of nasal epithelial cells from term infants. The results show that although neonatal cells exhibited a broader range of changes in protein expression than adult cells, yet the same set of proteins was significantly increased by EcO83 treatment compared to PBS (Figure S5). In conclusion, EcO83‐EVs consistently induce the expression of stress‐related proteins in both adult and newborn nasal epithelial cells (Figure S5).

### 3.4 EcO83‐EVs interact with airway cells in vivo

**FIGURE 5:**
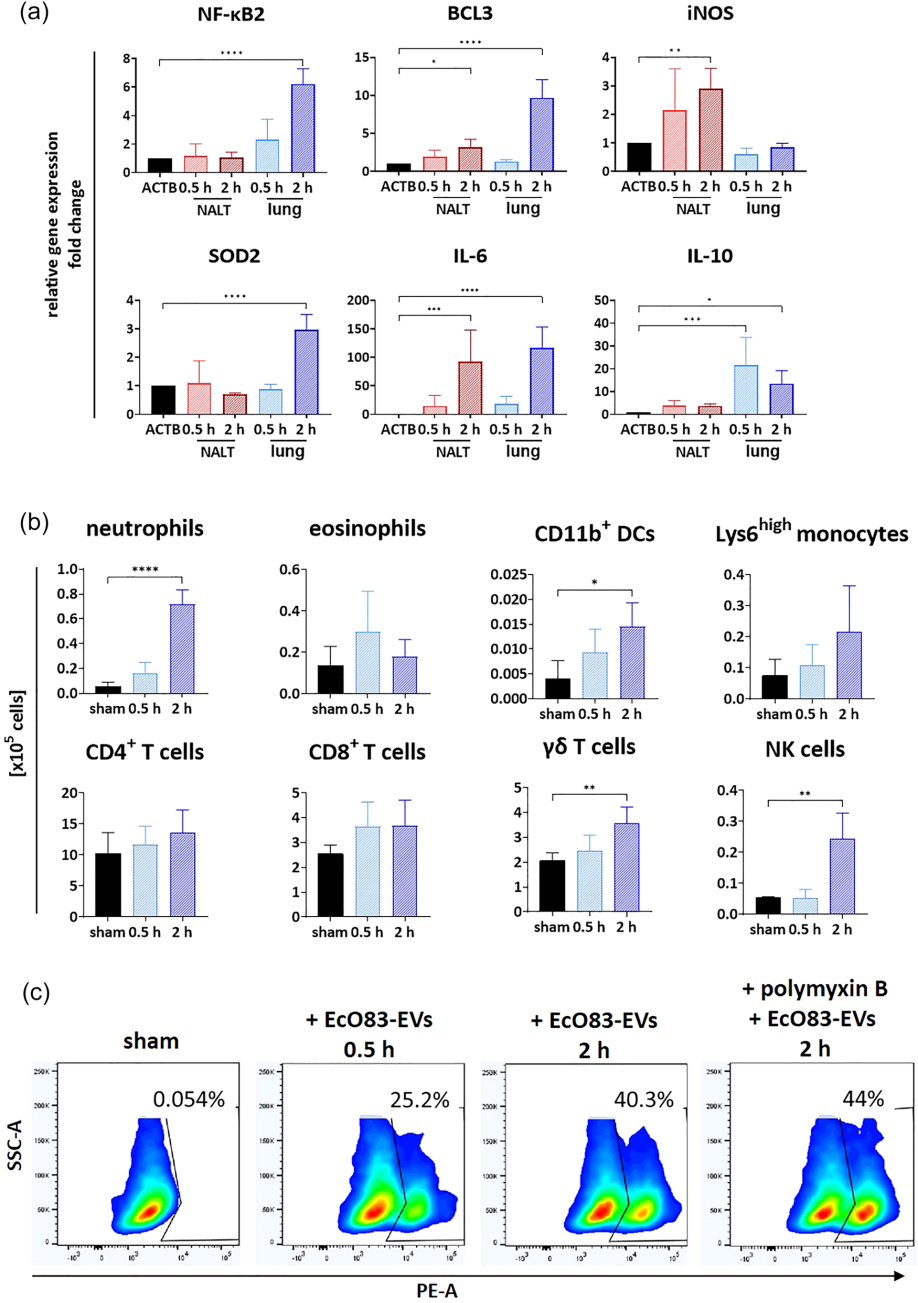
EcO83‐EVs interaction with mouse airways. Mice were administered rhodamine‐labelled or unlabelled 2.5 × 10^10^ EcO83‐EVs intranasally and sacrificed at either 0.5 h (*n *= 5) or 2 h (*n* = 5) post‐administration. Control mice (*n *= 3) were treated with 0.9% NaCl. The lungs and NALT were subsequently isolated for analysis. (a) Expression of specific genes at 0.5 and 2 h after treatment with unlabelled EcO83‐EVs analysed by qPCR. Reference gene α‐actin (ACTB) was used to normalize the gene expression data. (b) Cells recruited into lungs after 0.5 h or 2 h treatment with unlabelled EcO83‐EVs analysed by FACS. (c) FACS data obtained from lungs cells isolated from mice intranasally treated with rhodamine‐labelled EcO83‐EVs. Data shown for one representative experiment out of two. Statistical differences between samples were assessed using one‐way ANOVA; significance levels are denoted as *p ≤ 0.05, **p ≤ 0.01 ***p ≤ 0.001 ****p ≤ 0.0001 versus control.

Intranasal administration of EcO83‐EVs induced the expression of the BCL3, iNOS, and IL‐6 genes in the NALT and the NF‐κΒ2, BCL3, SOD2, IL‐6, and IL‐10 genes in the lung after 2 h (Figure 5a). The response was time‐dependent. IL‐10 gene expression in the lung reached a maximum 0.5 h after treatment with EcO83‐EVs and declined after 2 h. For IL‐6, maximal expression was observed both in the NALT and the lung 2 h after administration of EVs. At the cellular level, intranasal administration of EcO83‐EV induced the recruitment of neutrophils, CD11b+ dendritic cells, γδ T cells, and natural killer cells, to the lungs (Figure 5b). Using rhodamine B‐labelled EVs, we observed that EcO83‐EVs were internalised by lung cells and macrophages were primarily responsible for uptake (Figure 5c and S6). Notably, EcO83‐EVs uptake appears to occur independently of TLR4‐mediated signalling, as incubation of EcO83‐EVs with polymyxin B did not prevent their uptake by macrophages.

### 3.5 EcO83‐EVs induce NO in mice macrophages in a TLR4‐dependent manner

**FIGURE 6:**
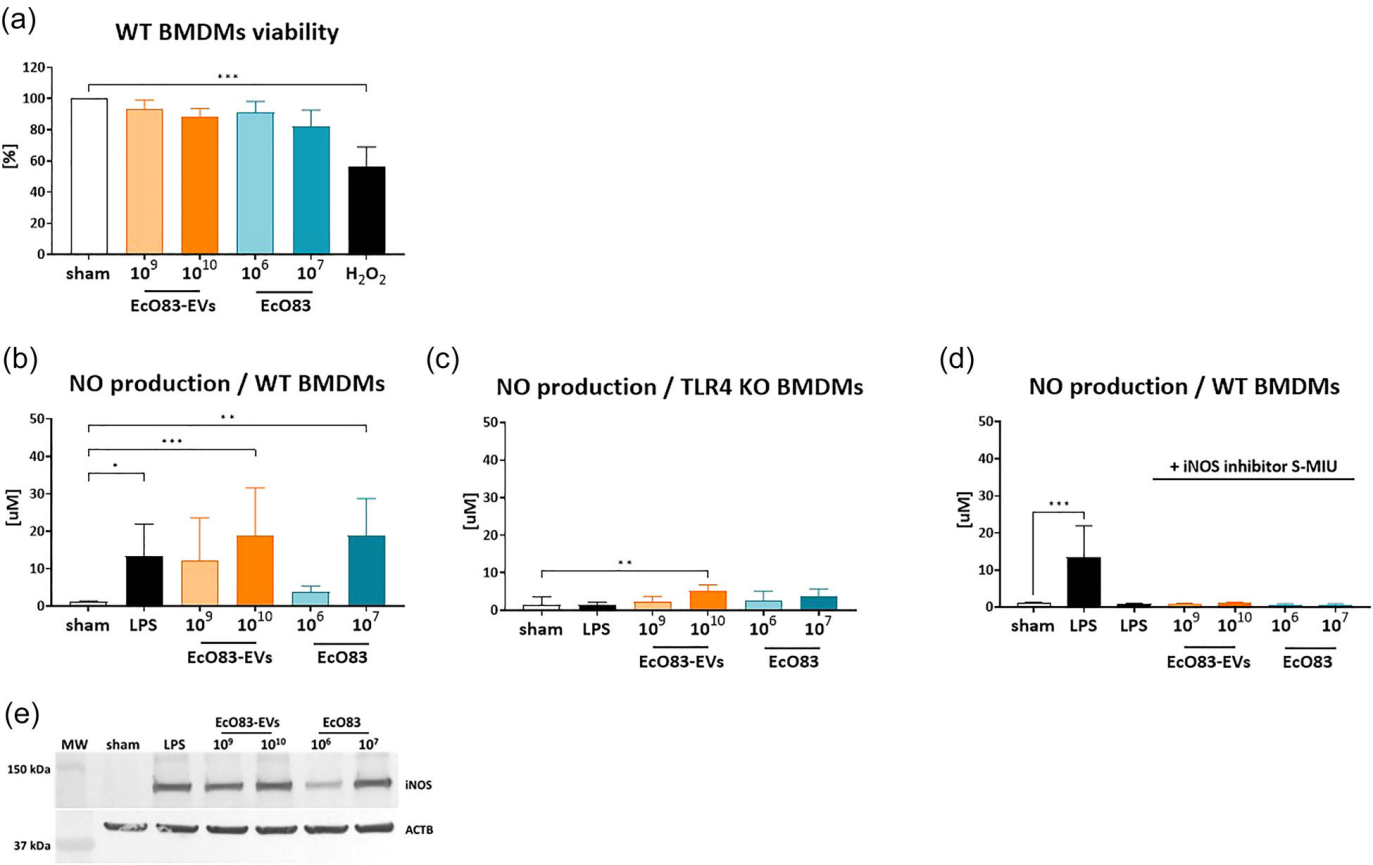
EcO83‐EVs interaction with BMDMs. (a) Viability of WT BMDMs when treated with 1 × 10^9^ and 1 × 10^10^ EcO83‐EVs/mL or 1 × 10^6^ and 1 × 10^7^ EcO83/mL for 24 h. (b) Production of NO in WT BMDMs when treated with 1 × 10^9^ and 1 × 10^10^ EcO83‐EVs/mL or 1 × 10^6^ and 1 × 10^7^ EcO83/mL for 24 h. (c) NO production in TLR4 KO BMDMs treated with 1 × 10^9^ and 1 × 10^10^ EcO83‐EVs/mL or 1 × 10^6^ and 1 × 10^7^ EcO83/mL for 24 h. (d) Production of NO in WT BMDMs when pre‐treated for 1 h with iNOS inhibitor (S‐MIU, 10 µM) and next treated with 1 × 10^9^ and 1 × 10^10^ EcO83‐EVs/mL or 1 × 10^6^ and 1 × 10^7^ EcO83/mL for 24 h. (e) iNOS expression in WT BMDMs treated with 1 × 10^9^ and 1 × 10^10^ EcO83‐EVs/mL or 1 × 10^6^ and 1 × 10^7^ EcO83/mL for 24 h, analysed by immunoblotting of cell lysates with monoclonal anti‐iNOS antibodies. Non‐treated cells (sham) were used as a negative control and LPS from *E. coli* O55B5 (1 µg/mL) as positive control for NO and iNOS induction. One‐way ANOVA was used to examine mean differences between samples. *p ≤ 0.05, **p ≤ 0.01 ***p ≤ 0.001 ****p ≤ 0.0001 versus control.

Given the preferential uptake of intranasally administered EcO83‐EVs by macrophages, we decided to investigate this interaction in more detail by using a mouse BMDM cell line. Initially, we assessed the impact of EcO83‐EVs and the parental bacteria on the viability of BMDMs using the MTT assay. The results showed that EcO83‐EVs and EcO83 were non‐toxic to the macrophages even at high concentration (Figure 6a). Macrophages in the airways have been shown to generate NO in response to bacterial components such as LPS (Lundberg & Weitzberg, 2022). We show that both EcO83‐EVs and EcO83 stimulated NO production in BMDMs in a dose‐dependent manner. Notably, 1 × 10^10^ EcO83‐EVs and 1 × 10^7^ EcO83 cells induced NO levels comparable to those elicited by 1 µg LPS (Figure 6b). Mock‐EVs did not induce NO (Figure S4e).

To characterise the role of specific TLRs in NO production induced by EcO83‐EVs, we quantified NO levels in BMDMs genetically deficient in TLR2, TLR4, TLR7, and TLR9 expression. Our results show a significant reduction in NO production exclusively in TLR4‐deficient BMDMs, although NO was still induced by the highest concentration of EcO83‐EVs, albeit reduced almost fourfold (Figure 6c). Significantly increased levels of NO induced by EcO83‐EVs were measured in cells with non‐functional TLR2, TLR7, and TLR9 signalling compared to WT cells (Figure S7a‐c).

To determine whether induction of NO by EcO83‐EVs in BMDMs is dependent on inducible NO synthase (iNOS), we used the specific inhibitor S‐MIU. Treatment with S‐MIU prior to exposure to EcO83‐EVs or EcO83 completely abolished NO production (Figure 6d). In this sense, we show that the expression of iNOS was increased in BMDMs treated with EcO83‐EVs or EcO83 (Figure 6e).

Finally, the effect of EcO83‐EVs on cytokine production was also investigated. BMDMs incubated with EcO83‐EVs and EcO83 for 24 h secreted IL‐6, TNF‐α, and IL‐10 in a dose‐dependent manner (Figure S8a‐c).

### 3.6 EcO83‐EVs induce MAPK/NF‐κΒ signalling in mice macrophages

Given our findings that NO induction by EcO83‐EVs is dependent on TLR4, we further explored the downstream signalling pathways involved and compared these with the parent bacterium. As previously mentioned, NO synthesis is catalysed by iNOS, which expression is regulated by NF‐κΒ following activation by MAP kinases (MAPKs) (Dorrington & Fraser, 2019). Therefore, we investigated the effect of EcO83‐EVs and EcO83 on MAPKs ERK1/2 and JNK, and on the subsequent activation of NF‐κΒ by Western blot. The results show that both EcO83‐EVs and EcO83 significantly enhance the phosphorylation of ERK1/2 and JNK. However, the kinetics of these responses varied markedly (Figure 7). EcO83‐EVs induced early and sustained phosphorylation of ERK1/2, whereas EcO83 induced early but transient phosphorylation (Figure 7b and h). For JNK, EcO83‐EVs induced early and sustained activity, in contrast to the delayed response to the parent bacterium (Figure 7d and j). Accordingly, NF‐κΒ phosphorylation was rapidly and transiently initiated in response to EcO83‐EVs, whereas stimulation with EcO83 elicited a delayed and sustained response (Figure 7f and l). By pre‐incubation with specific inhibitors, we confirmed the involvement of ERK1/2 and JNK in NO production by EcO83 and its EVs (Figure 7c, e, i, k).

FIGURE 7EcO83‐EVs and EcO83 effect on the MAPK and NF‐κΒ transcription factor activation in BMDM cells. (a) Phosphorylation status of ERK1/2, JNK, and p65 proteins in BMDMs treated with 1 × 10^10^ EcO83‐EVs/mL for 15–120 min analysed with immunoblotting. (b) Densitometry analysis of p‐ERK1/2/ERK1/2 in BMDM cells treated with 1 × 10^10^ EcO83‐EVs/mL for 15–120 min. (c) NO production of BMDM cells treated with 1 × 10^10^ EcO83‐EVs/mL for 24 h with/without 1 h pre‐treatment of ERK1/2 inhibitor (20 µM of U0126). (d) Densitometry analysis of p‐JNK/JNK in BMDM cells treated with 1 × 10^10^ EcO83‐EVs/mL for 15–120 min. (e) NO production of BMDM cells treated with 1 × 10^10^ EcO83‐EVs/mL for 24 h with/without 1 h pre‐treatment of JNK inhibitor (10 µM of SP600125). (f) Densitometry analysis of p‐p65/NF‐κΒ in BMDM cells treated with 1 × 10^10^ EcO83‐EVs/mL for 15–120 min. (g) Phosphorylation status of ERK1/2, JNK and p65 proteins in BMDMs treated with 1 × 10^7^ EcO83/mL for 15–120 min analysed with immunoblotting. (h) Densitometry analysis of p‐ERK1/2/ERK1/2 in BMDM cells treated with 1 × 10^7^ EcO83/mL for 15–120 min. (i) NO production of BMDM cells treated with 1 × 10^7^ EcO83/mL for 24 h with/without 1 h pre‐treatment of ERK1/2 inhibitor (20 µM of U0126; the upstream kinase of p42 and p44 ERK1/2). (j) Densitometry analysis of p‐JNK/JNK in BMDM cells treated with 1 × 10^7^ EcO83/mL for 15 ‐ 120 min. (k) NO production of BMDM cells treated with 1 × 10^7^ EcO83/mL for 24 h with/without 1 h pre‐treatment of JNK inhibitor (10 µM of SP600125). (l) Densitometry analysis of p‐p65/NF‐κΒ in BMDM cells treated with 1 × 10^7^ EcO83/mL for 15–120 min. One‐way ANOVA was used to examine mean differences between samples. *p ≤ 0.05, **p ≤ 0.01 ***p ≤ 0.001 ****p ≤ 0.0001 versus control.

**Figure:**
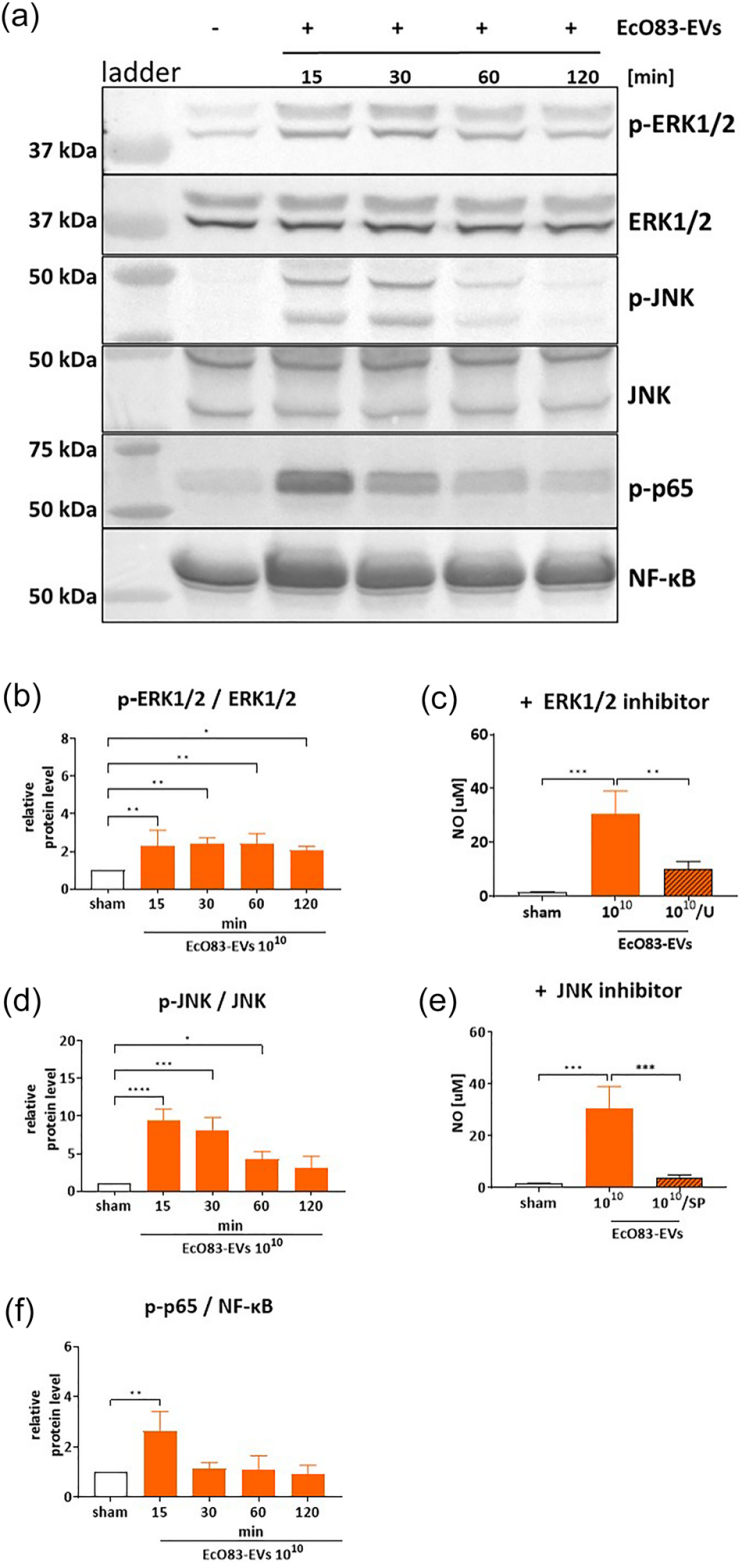

**Figure:**
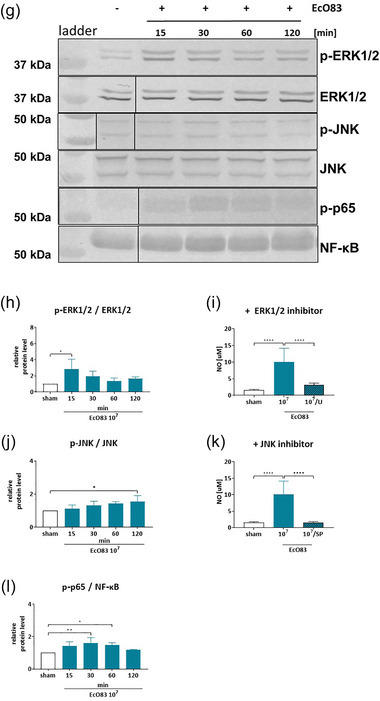

In comparison, we analysed the effects of LPS from *E. coli* O55B5 on these signalling pathways (Figure S9). Following LPS treatment, there was a notable increase in ERK1/2 phosphorylation after 60 min, significantly later than the response to EcO83‐EVs. Conversely, the rapid increase in JNK and NF‐κB phosphorylation mirrored the activation observed with EcO83‐EVs, suggesting that the pattern of LPS activation of the MAPK/NF‐κB signalling pathways is more similar to that of EcO83‐EVs than that of the parent bacterium.

## 4 DISCUSSION

Experimental studies in animals, as well as research involving human subjects, have demonstrated that the probiotic bacterium *E. coli* O83 possesses potent immunomodulatory properties (Lodinová‐Zádníková et al., 2003; Lodinová‐Zádníková et al., 1995; Lodinová‐Žádníková et al., 2010; Súkeníková et al., 2017; Súkeníková et al., 2022). For example, early postnatal oral administration of the live bacteria led to stable colonisation and reduced incidence of nosocomial infections in newborns (Lodinová‐Zádníková et al., 1995). Although probiotics are generally regarded as safe, their use is not without risks and adverse effects have been reported (Kothari et al., 2019), emphasising the need for innovative safe alternatives.

Bacterial EVs have shown promise as postbiotics (Xie et al., 2024) and as a new generation of mucosal vaccines (Peng et al., 2021). The use of bacterial EVs may address several concerns associated with live probiotics, such as the risk of transferring antibiotic resistance, the potential to cause sepsis in immunocompromised individuals and the need to maintain viability in probiotic formulations (Kothari et al., 2019; Trush et al., 2020). Despite these advantages, extensive research is required to understand the mechanism of interaction between bacterial EVs and the host before they reach the clinics. In this work, we characterise EcO83‐EVs and their interactions with the respiratory tract in detail using human epithelial cells and mouse models.

LPS is a specific component of G‐negative bacteria and a potent inducer of immune responses. In particular, LPS isolated from the probiotic *E. coli* Nissle 1917 and the uropathogenic *E. coli* W536 has been shown to induce high levels of IL‐10 in human peripheral blood mononuclear cells (PBMCs) (Güttsches et al., 2012). Remarkably, we demonstrated that LPS is the most abundant component in EcO83‐EVs with a concentration of about 1 mg per 1 × 10^11^ EcO83‐EVs, which is about 15‐fold of the total protein content. Previous studies by our group have shown that the immunomodulatory effects of EcO83 bacteria depend on signalling through TLR4, implying that EcO83 LPS may contribute to the probiotic properties of this bacterial strain (Schmid et al., 2023; Zwicker et al., 2018). Here we show that EcO83‐EVs increased the NO production and iNOS expression in macrophages and that the NO production was, at least partly, dependent on TLR4. Importantly, we have demonstrated that the mock‐EVs, an essential control, do not induce NO production, in contrast to EcO83‐EVs. NO is a molecule that has antimicrobial, antioxidant and anti‐inflammatory properties (Sharma et al., 2007). NO is released and mediated by iNOS, which is expressed in cells involved in anti‐inflammatory processes (Andrabi et al., 2023). For example, activated airway macrophages express high levels of iNOS and produce high levels of NO leading to the elimination of intracellular microbial pathogens in the airways (Bayarri et al., 2021). Similarly, EVs from *E. coli* Nissle 1917 induced iNOS and NO production in macrophages, which was associated with enhanced antibacterial activity of these cells (Hu et al., 2020).

To link the host immune response to EcO83‐EV cargo and to determine the origin of these EVs, we conducted several quantitative and qualitative analyses. These tests compared the protein, lipid, DNA and RNA composition of the EVs with that of their parent bacteria. The results show that EcO83‐EVs are particularly enriched in membrane and outer membrane proteins, similar to what has been reported for the protein composition of EVs from another *E. coli strain* (DH5α) (Lee et al., 2007). We also show that the surface lipids are almost identical between the EVs and the bacteria. Furthermore, the ZP values of the EcO83‐EVs and the parent bacteria are also very similar, providing insight into the surface charge and colloidal stability and supporting the similarities in surface chemistry between the EVs and the bacteria (Midekessa et al., 2020). Combining these observations with our findings that DNA was absent in the EVs, we speculate that EcO83‐EVs originate from their parent bacteria by blebbing of the outer membrane, as discussed by Toyofuku et al. (Toyofuku et al., 2023). However, this hypothesis is challenged by the presence of RNA, a typical cytosolic component, inside the EcO83‐EVs, despite treatment with proteinase and RNAse, which removes the externally attached RNA. Therefore, the exact biogenesis of EcO83‐EVs still needs to be investigated.

The RNA that we isolated from the EcO83‐EVs, contained traces of 23S and 16S rRNA and was enriched in small RNA. EV‐associated RNA has been proposed as a universal mediator for inter‐bacterial communication and microbiota‐host interaction, as it can interfere with protein translation (Munhoz da Rocha et al., 2020). Whether EcO83‐EVs are capable of transporting their RNA cargo into host cells, as was shown for *E. coli* 536 EVs (Blenkiron et al., 2016) remains to be investigated.

The mass ratio of proteins to lipids in EcO83‐EVs is approximately 1:1. This ratio is consistent with that reported for *Bacteroides thetaiotaomicron* EVs collected in the early phase of culture (Juodeikis et al., 2024), and which also varies depending on the bacterial growth phase, with cytoplasmic proteins being favoured in the late phase. Differences in proteomic profiles were also reported for *Staphylococcus aureus* EVs collected at early and late stages of bacterial culture (da Luz et al., 2022). Therefore, it is crucial to document the culture conditions—especially, the culture time—when reporting such data. Ongoing studies are investigating the influence of culturing conditions, including time and growth media stress, on the cargo profiles of EVs.

The ability of EVs to bind molecules present in the environment, such as cell debris or components of the culture medium, is well–documented (Buzás et al., 2018). To ensure that the biological effects are solely attributed to EVs, rigorous purification steps are essential. We performed UC, leading to so‐called crude EVs, followed by DG‐UC, a widely accepted technique in EV research, to reduce potential non‐vesicular contamination (Narciso & Aschtgen, 2023). Despite this additional step, fibrillary structures were observed in the EV preparations by AFM. Proteomic analyses of EcO83‐EVs, both before and after DG purification, revealed an enrichment of fimbrial proteins after DG‐UC. It is known that *E. coli* bacteria produce two fibrillary structures: fimbriae and flagella, with densities of 1.1 g/mL for fimbriae isolated from *E. coli* WF96 (Karch et al., 1985) and approximately 1.3 g/mL for flagella isolated from *E. coli* K‐12 (DePamphilis & Adler, 1971). The density of EcO83‐EVs, which ranges from 1.190 to 1.267 g/mL, suggests potential co‐purification with fimbrial proteins. This observation emphasises the need for further investigation into the fibrillary structures of the EcO83 strain and highlights the critical impact of the purification method on the final EV composition.

Given their immunomodulatory properties, probiotics have been extensively explored as carriers and adjuvants for mucosal vaccination and immunotherapy. In our previous studies, we have studied *E. coli* Nissle 1917 and lactic acid bacteria as delivery vectors for allergens in allergen‐specific intranasal immunotherapy (Daniel et al., 2006; Sarate et al., 2019). Furthermore, we have shown that intranasal administration of either EcO83 or EcO83‐EVs prevents the development of allergic asthma in mouse model (Schmid et al., 2023; Zwicker et al., 2018). Extending these findings, we examined the mechanism of interaction between EcO83‐EVs and healthy host airway cells both in vitro and in vivo. Proteomic analysis of primary epithelial cell cultures isolated from adult human subjects treated with EcO83‐EVs revealed induction of inflammatory and stress responses, notably upregulating CXCL8 ‐ a chemokine that recruits neutrophils, basophils and T cells in response to pathogens (Hammond et al., 1995). Correspondingly, a significant influx of neutrophils was observed in the lungs of mice 2 h post‐intranasal administration of EcO83‐EV. Given the initial use of EcO83 in newborns (Kocourková et al., 2007) we further explored whether similar responses were elicited in epithelial cells isolated from this age group. Although a broader spectrum of protein expression alterations was noted in neonates compared to adults, the most significantly upregulated proteins were consistent across both groups. Interestingly, neonatal cells exhibited a downregulation in tubulin‐related proteins (e.g. TUBB4A, TUBB2A, TUBB6) and an upregulation in inflammation‐related proteins (e.g. CXCL1, TNFAIP2 and 3, PTGS2), underscoring the distinctive functional capacities of neonatal versus adult airway epithelia. Neonatal nasal airways, richer in polymorphonuclear leukocytes and granulocytes, are predisposed to more severe pathogen‐induced inflammatory responses, manifested by elevated levels of induced peroxides and inflammatory cytokines (Futata et al., 2012). These physiological differences are critical considerations in the design of probiotic treatments tailored to adults and children, impacting their therapeutic efficacy.

In adult nasal epithelial cells differentiated at the air‐liquid interface culture, a modest upregulation of proteins associated with inflammation and stress was observed, notably SOD2, NF‐κΒ2, and BCL3. This enhancement in gene expression was consistent across different models and was mirrored in lung tissues of mice treated intranasally with EcO83‐EVs, where an increase in expression of the same inflammation‐associated proteins, as well as IL‐6 and IL‐10, was demonstrated. Of note, the expression of IL‐10 reached the maximum within 30 min post‐administration of EcO83‐EVs and subsequently decreased over a 2‐h period. The critical role of IL‐10 in modulating airway inflammation is underscored by its ability to suppress the production of pro‐inflammatory cytokines and chemokines (Peñaloza et al., 2015). Our previous studies have shown that intranasal administration of probiotic bacteria induced IL‐10 production in vivo, which was associated with beneficial effects in a mouse model of allergic inflammation (Schabussova et al., 2011). Additionally, our data suggest a similar cytokine response to EcO83‐EV exposure across several cell types, including mouse lung cells, human epithelial cells, human PBMCs, and monocyte‐derived dendritic cells (unpublished data). However, the precise cellular source of IL‐10 in this context remains to be identified.

A limitation of our study is the use of different metrics for determining the dose of EcO83‐EVs in ex vivo versus in vivo and in vitro experiments. Initially, we used protein concentration to determine EV doses for ex vivo experiments. However, to achieve better reproducibility and accuracy, we later switched to using particle numbers for in vivo and in vitro experiments. Due to the limited number of patient samples and the complexity of the culture procedure, we were unable to repeat the *ex vivo* experiments using the vesicle numbers.

To further investigate the interaction between EcO83‐EVs and lung cells, we performed an uptake study with rhodamine‐labelled EcO83‐EVs. It is important to note that in the EV field, there is a great variety of methods used for EV labelling (Loconte et al., 2023). For our studies, we opted for rhodamine B, a lipophilic, self‐quenched dye which fluorescence intensifies after fusion with membranes (Montecalvo et al., 2012), serving as an indirect marker of EVs uptake. Following the labelling process, we used a bottom‐up DG purification to efficiently separate labelled EVs from any residual dye remaining in the bottom fraction of the DG (Deville et al., 2021). Recognising the inherent limitations associated with lipophilic dyes for EV staining, we increased the dilution of rhodamine to prevent the formation of dye aggregates (data not shown). As an additional control, we performed an experiment with a rhodamine B dye processed in the same way as the labelled EcO83‐EVs. FACS analysis of the lung cells showed no labelling in any cell population (data not shown), confirming the specificity of EcO83‐EV uptake by macrophages. To improve the biodistribution studies, we plan to genetically engineer EcO83 and use the SpyTag/Catcher system (Brune et al., 2016).

Given the potential role of macrophages as the primary source of early‐induced IL‐10 and their unique capacity to internalize EcO83‐EVs upon intranasal delivery, we focused on elucidating the signalling pathways activated by EcO83‐EVs in these cells. For our experiments, we utilized the BMDM cell line, which provides a homogeneous macrophage population. Under homeostasis, macrophages patrol the lung environment to identify potential threats, produce IL‐10 and eliminate pathogens by phagocytosis without triggering an adaptive immune response. In case of danger, they polarize towards a pro‐inflammatory M1 phenotype, characterised by elevated production of NO, TNF‐α, and IL‐6, which are all key mediators in immune regulation and inflammatory responses (Atri et al., 2018). When stimulated with EcO83‐EVs, macrophages demonstrated a dual response, expressing both pro‐inflammatory TNF‐α and anti‐inflammatory IL‐10, alongside the pleiotropic cytokine IL‐6. This immunological profile aligns with studies on EVs from other *E. coli* probiotic strains (Fábrega et al., 2016; Wan et al., 2018).

Phosphorylation of MAPKs initiates the activation of NF‐κΒ, a pivotal regulator of many genes integral to the innate immune response, including those coding TNF‐α and IL‐6, as well as iNOS (Dorrington & Fraser, 2019; Salim et al., 2016). Therefore, we investigated the effect of EcO83‐EVs on activation of these signalling pathways. Our data show that EcO83‐EVs induced early and sustained activation of ERK 1/2 and JNK, in contrast to early, but transient phosphorylation of ERK 1/2 and delayed phosphorylation of JNK by EcO83 bacteria. Notably, the early ERK 1/2 activation by EcO83‐EVs observed at 15 min post‐stimulation, may activate macrophages and lead to the production of inflammatory mediators such as TNF‐α or NO (Valledor et al., 2000). Conversely, the later activation of JNK by EcO83, unlike with EcO83‐EVs, may enhance antigen presentation since it has been suggested that JNK‐1 orchestrates the regulation of genes involved in antigen presentation (Valledor et al., 2008). Further studies are needed to delineate these differences, and their potential biological consequences comprehensibly. We hypothesise that due to their smaller size, EcO83‐EVs might more rapidly reach the target (easier and faster taken up by cells) and elicit immunomodulatory effects compared to parent bacteria. Thus, production of EVs appears to be another mechanism used by the bacterium to modulate the conditions of the surrounding environment.

In summary, we have established a method for the isolation, purification and characterisation of probiotic EcO83‐EVs. EcO83‐EVs retain characteristics similar to those of their parent bacteria, yet they introduce entirely new possibilities. Notably, they are non‐toxic and exhibit immunomodulatory properties which opens the way to use them as intranasal postbiotics. Moreover, they have adjuvant effects, which, in combination with appropriate antigens, may allow them to be used as convenient mucosal vaccines.

## AUTHOR CONTRIBUTIONS

**Agnieszka Razim**: Formal analysis (equal); investigation (lead); methodology (equal); writing—original draft (lead); writing—review and editing (equal). **Agnieszka Zabłocka**: Investigation (equal); methodology (equal); writing—original draft (equal); writing—review and editing (supporting). **Anna Schmid**: Investigation (supporting); methodology (equal); writing—original draft (supporting); writing—review and editing (supporting). **Michael Thaler**: Investigation (supporting). **Viktor Černý**: Investigation (supporting); writing ‐ original draft (supporting). **Tamara Weinmayer**: Investigation (supporting). **Bradley Whitehead**: Data curation (equal); investigation(equal); Writing ‐ review & editing (supporting). **Anke Martens**: Data curation (equal); formal analysis (supporting); investigation (equal); methodology (equal); writing—original draft (supporting). **Magdalena Skalska**: Data curation (equal); investigation (equal); methodology (equal); writing—original draft (supporting). **Mattia Morandi**: Investigation (supporting); writing—review and editing (supporting). **Katy Schmidt**: Investigation (supporting); writing—original draft (supporting). **Magdalena E. Wysmołek**: Investigation (supporting); methodology (supporting). **Akos Végvári**: Data curation(equal); investigation (equal); methodology (equal). **Dagmar Srutkova**: Conceptualization (supporting); investigation (supporting); writing—original draft (supporting). **Martin Schwarzer**: Conceptualization (supporting); investigation (supporting); writing—original draft (supporting). **Lukas Neuninger**: Investigation (supporting). **Peter Nejsum**: Investigation (supporting); writing—review and editing (supporting). **Jiri Hrdý**: Investigation(supporting); writing ‐ review & editing|Supporting. **Johan Palmfeldt**: Investigation (equal); methodology (equal); writing—review and editing (supporting). **Marco Brucale**: Investigation (supporting); writing—review and editing (supporting). **Francesco Valle**: Investigation (supporting); writing—review and editing (supporting). **Sabina Górska**: Conceptualization (equal); funding acquisition (equal); methodology (equal); resources (equal); writing—review and editing (equal). **Lukas Wisgrill**: Data curation (equal); investigation (equal); methodology (equal); writing—review and editing (supporting). **Aleksandra Inic‐Kanada**: Formal analysis (equal); methodology (supporting); writing—review and editing (equal). **Ursula Wiedermann**: Funding acquisition (supporting); Resources (equal); writing ‐ review & editing|Supporting. **Irma Schabussova**: Conceptualization (lead); funding acquisition (lead); resources (equal); writing—original draft (equal); writing—review and editing (equal).

## CONFLICT OF INTEREST STATEMENT

The authors report no conflict of interest.

## ETHICS APPROVAL STATEMENT AND PATIENT CONSENT STATEMENT

Experiments were approved by the Animal Experimentation Committee of the Medical University of Vienna and the Austrian Federal Ministry of Education, Science and Culture (BMBWF‐66.009/0277‐V/3b/2019). The study was approved by the ethics committee of the Medical University of Vienna (EK 2164/2017). Both parents and adult participants gave signed written informed consent in accordance with the Declaration of Helsinki prior to enrolment in the study.

## Supporting information



Supporting Information

## Data Availability

Authors of the paper deposited the following data in open repositories: EVs and cellular MS proteomics data have been deposited to the ProteomeXchange Consortium via the PRIDE partner repository with the dataset identifier PXD052178 and PXD052553, respectively. Lipidomics was deposited in the RODBUK—Cracow Open Research Data Repository (doi.org/10.57903/UJ/RLIHE7). Other data will be available upon a reasonable request.
