# Supplementary material for: Bacterial extracellular vesicles as intranasal postbiotics: Detailed characterization and interaction with airway cells
Source: J Extracell Vesicles. 2024 Oct 21;13(10):e70004. doi: 10.1002/jev2.70004 (PMC11491762; doi:10.1002/jev2.70004)
Supplement: Supplementary file 1 — Supporting Information [file JEV2-13-e70004-s001.docx]

**SUPPLEMENTARY MATERIAL**

**Figure S1**


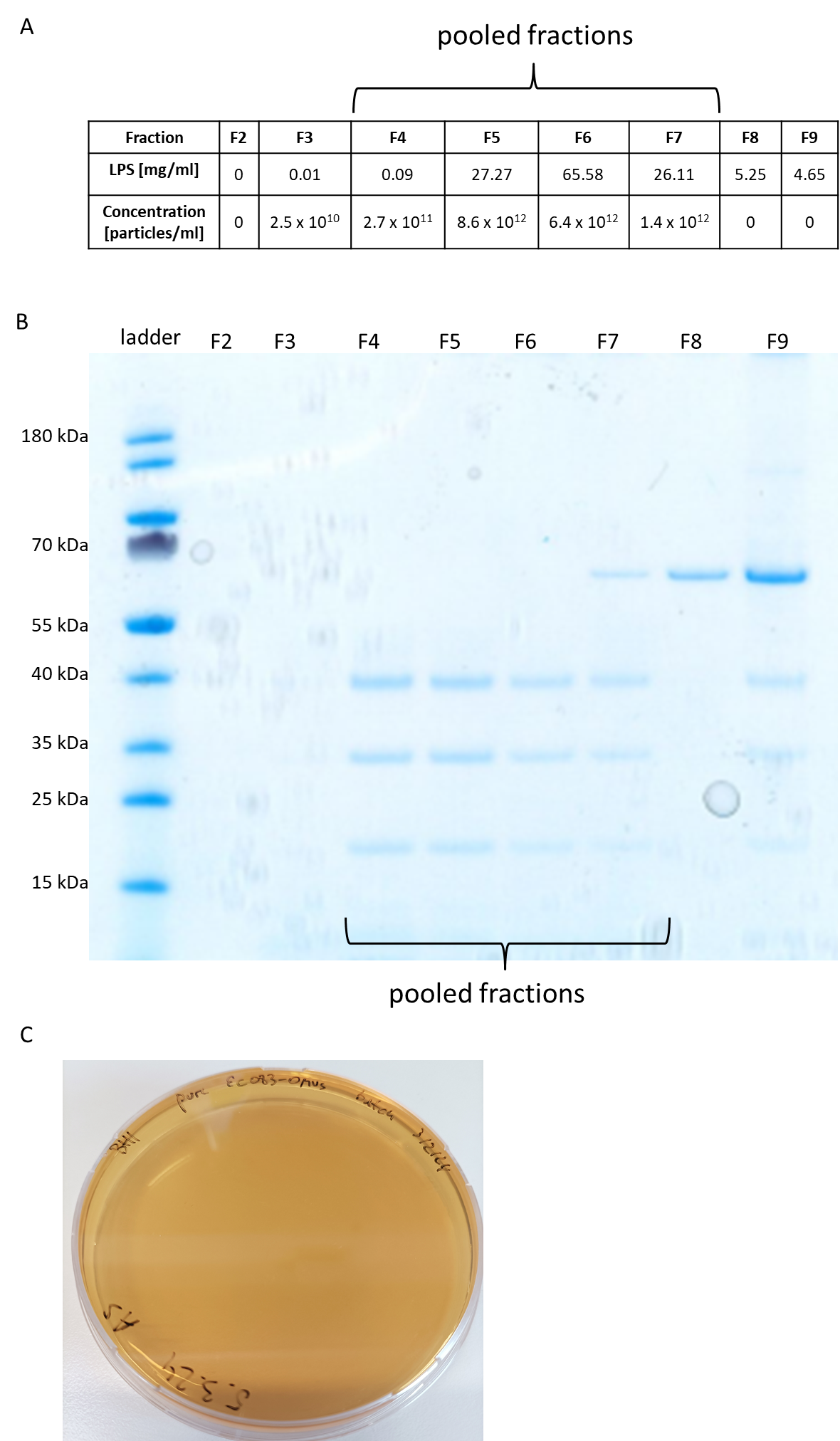


**Characterisation of fractions of EcO83-EVs collected after density gradient ultracentrifugation.** **(A)** LPS content determined by the LAL assay and particle concentration assessed by Zetasizer. **(B)** Protein profile of the individual fractions analysed by SDS-PAGE. **(C)** Sample of EcO83-EVs (pooled fractions F4-F7) plated on a BHI agar plate.

**Figure S2**


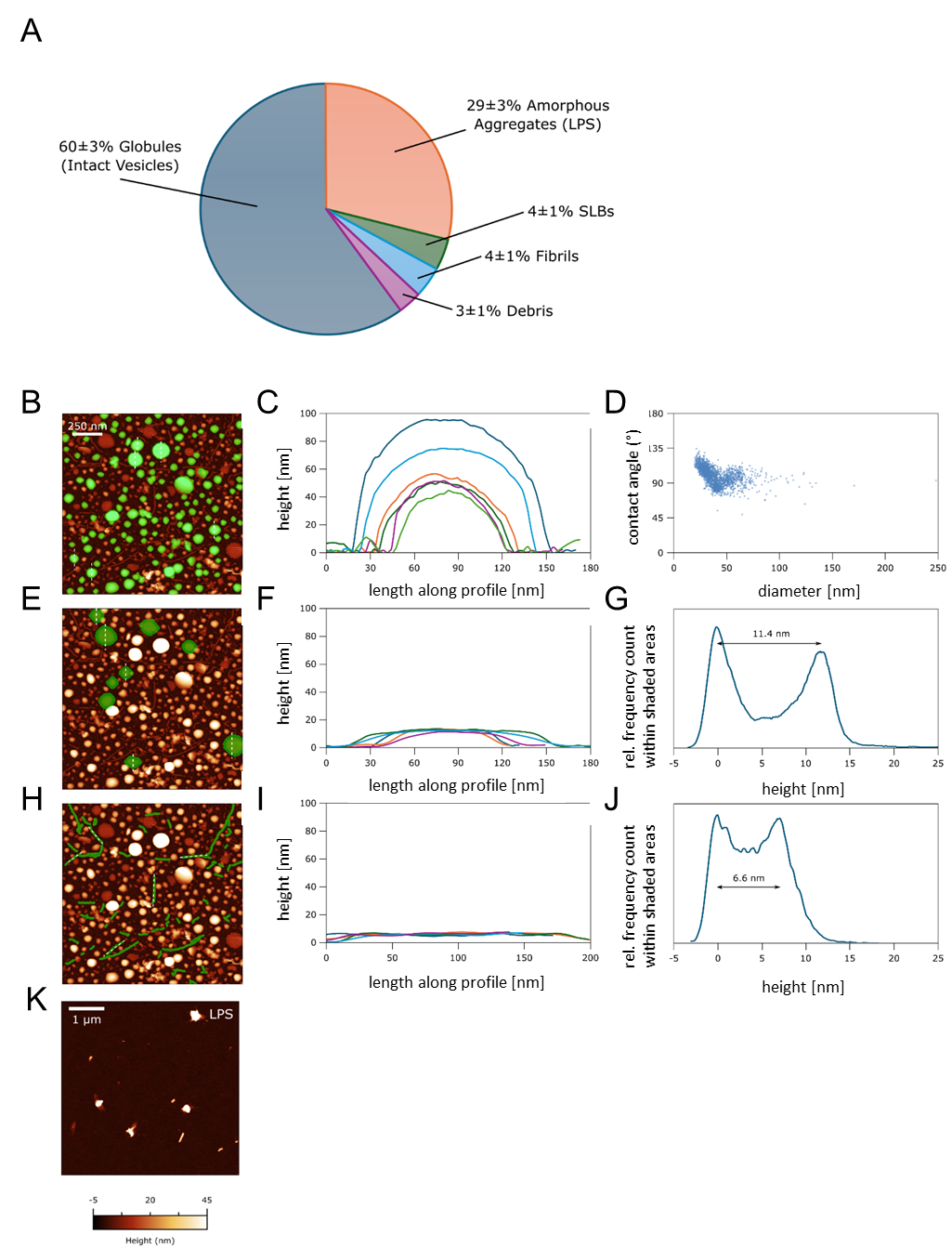


**Analysis of EcO83-EVs by atomic force microscopy.** **(A)** Quantification of the different structures found in the EcO83-EVs sample, expressed as % of the total volume of objects larger than 4 nm. **(B)** Representative atomic force microscopy (AFM) micrograph with indicated intact EcO83-EVs (in green). **(C)** Height profiles of intact EcO83-EVs indicated in B. **(D)** AFM morphometry of individual intact EcO83-EVs (n = 1760). Each particle is assigned a contact angle and a diameter. **(E)** Representative AFM micrograph with indicated burst EcO83-EVs (in green). **(F)** Height profiles of the burst EcO83-EVs measured along the dashed lines in E. **(G)** Height distribution measured in the green areas in E. **(H)** Representative AFM micrograph of fibrillar objects. **(I)** Height profiles of fibrillary objects measured along the dashed lines in H. **(J)** Height distribution measured in the green areas in H. **(K)** Representative AFM micrograph of LPS.

**Figure S3**


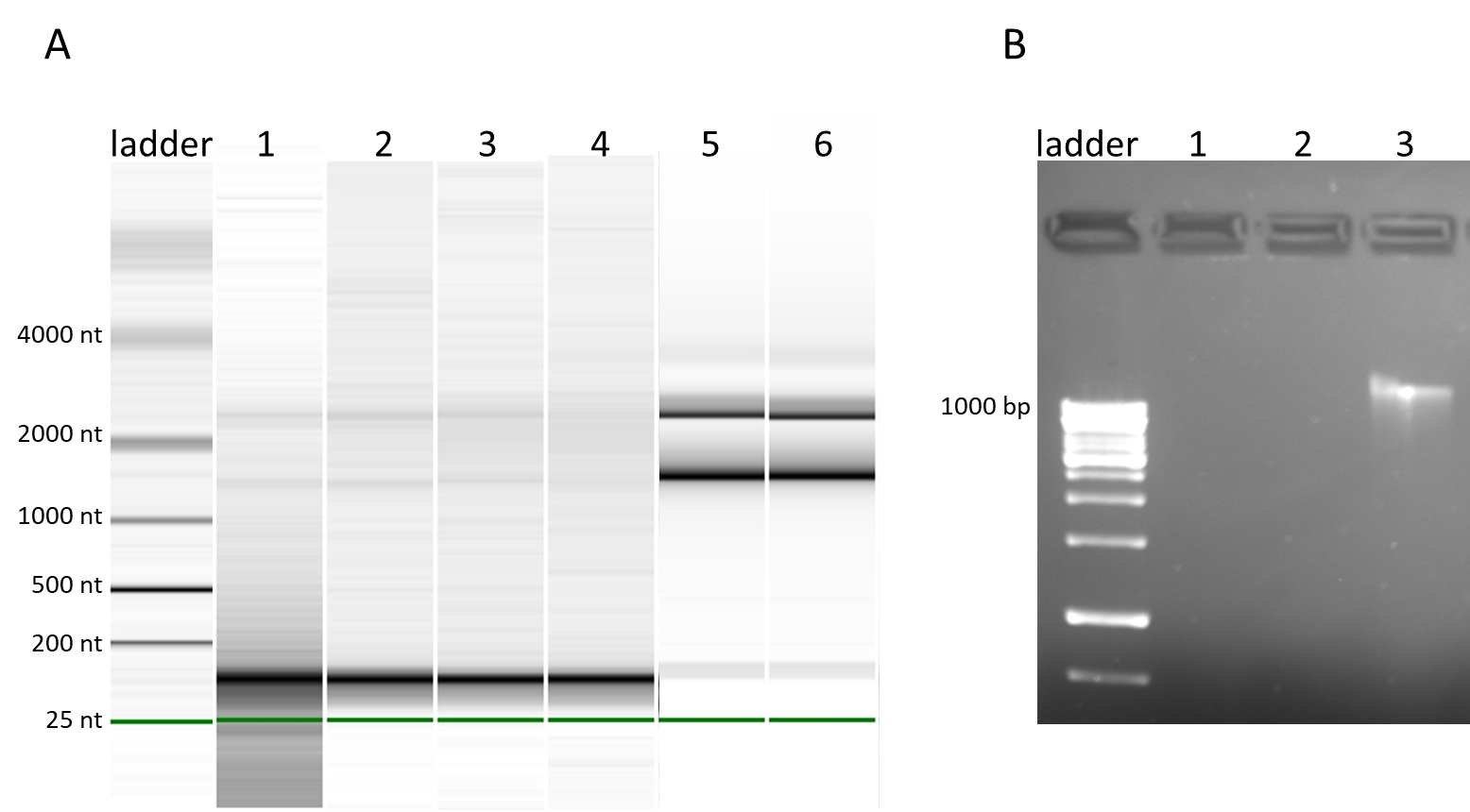


**Measurement of RNA and DNA in ECO83-EVs and EcO83. (A)** RNA isolated from EcO83-EVs (lines 1-2), EcO83-EVs pre-treated with proteinase K and RNAse (lines 3-4), and EcO83 (lines 5-6). **(B)** Total DNA isolated from EcO83-EVs (line 1), EcO83-EVs pre-treated with proteinase K and DNAse (line 2) and EcO83 (line 3).

**Figure S4**


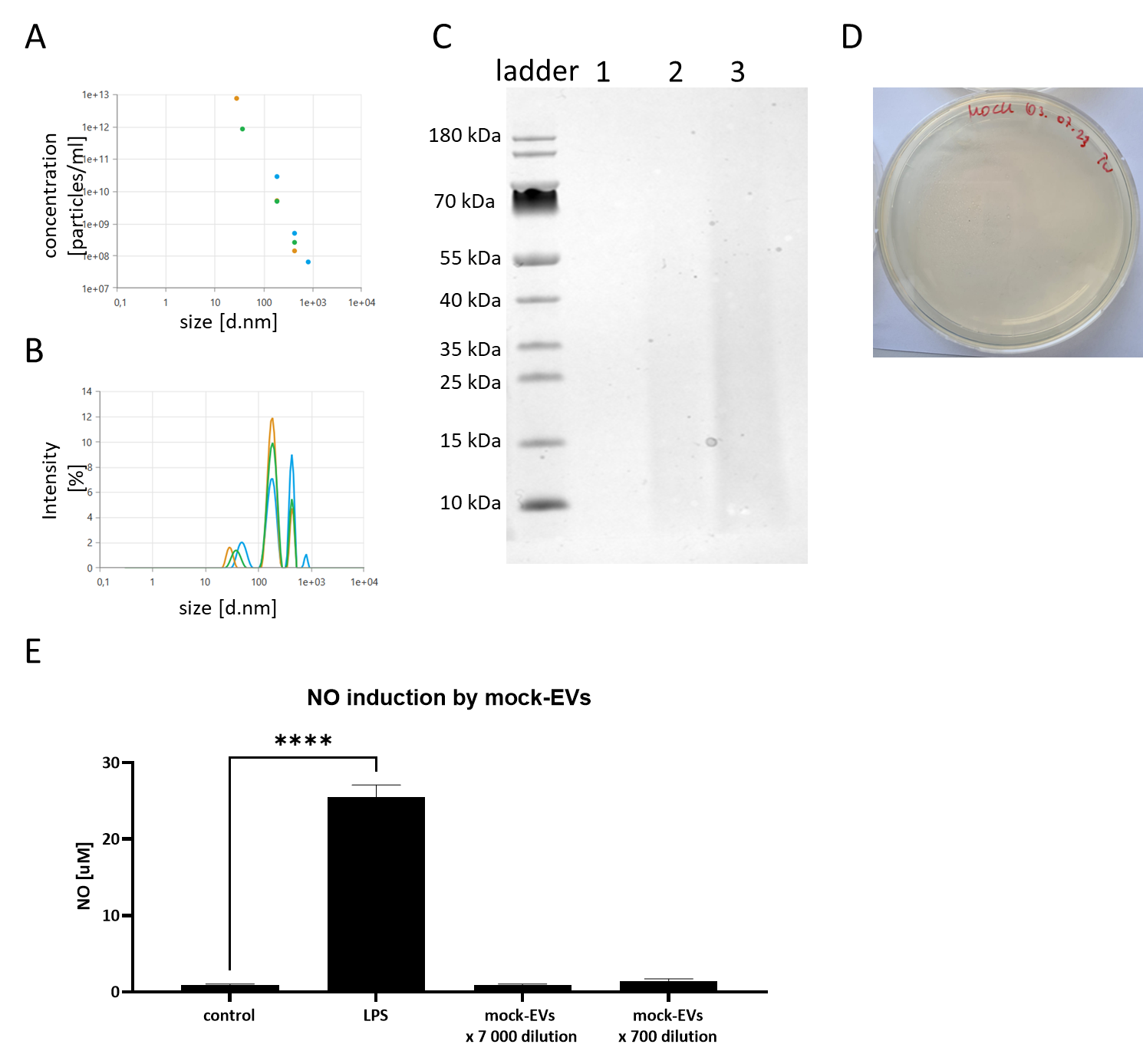


**Characterization of mock-EVs.** BHI medium without bacteria was ultracentrifuged to obtain mock-EV control. **(A)** Particle concentration in mock-EVs assessed by using a Zetasizer. **(B)** Particle size measurement of mock-EVs by using Zetasizer. **(C)** Protein profile of mock-EVs analysed by SDS-PAGE. 1 µl (line 1), 5 µl (line 2) and 10 µl (line 3) of mock-EVs were loaded on the gel. **(D)** Sample of mock-EVs plated on a BHI agar plate. **(E)** Production of NO in wild type bone marrow-derived macrophages treated with media (control), LPS (1 µg/ml) and mock-EVs (the same dilutions used as in corresponding experiments with EcO83-EVs) for 24 hours.

**Figure S5**

**
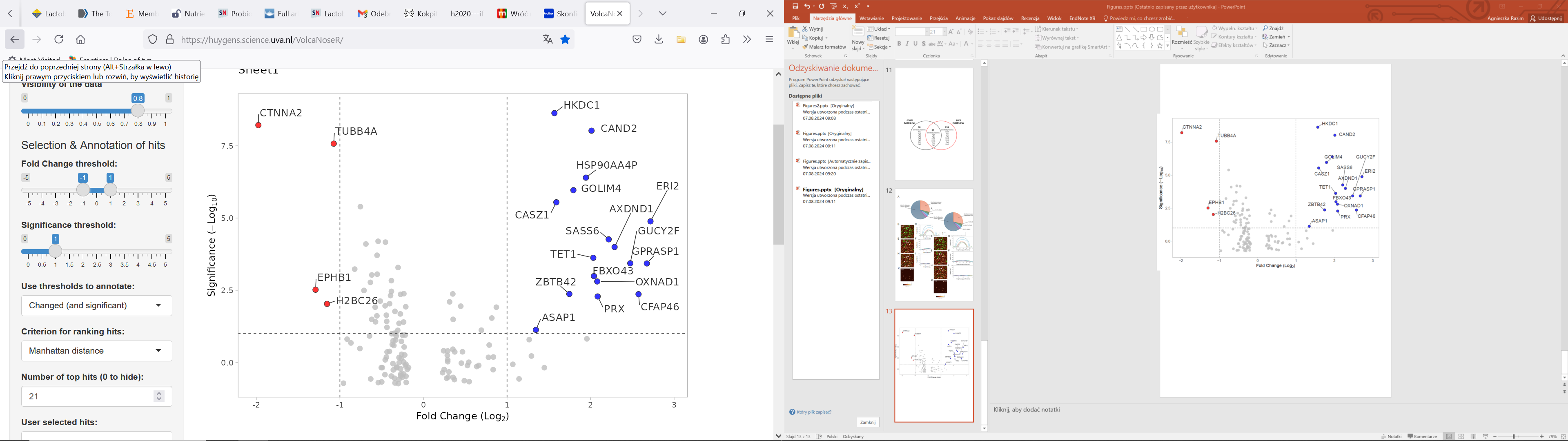
**

**Proteomics of term newborns nasal epithelial cells treated with EcO83-EVs ex vivo.** **(A)** Volcano plot of differentially expressed proteins for term infants air-liquid interface cell cultures after 24 h incubation with EcO83-EVs and PBS.

**Figure S6**

**
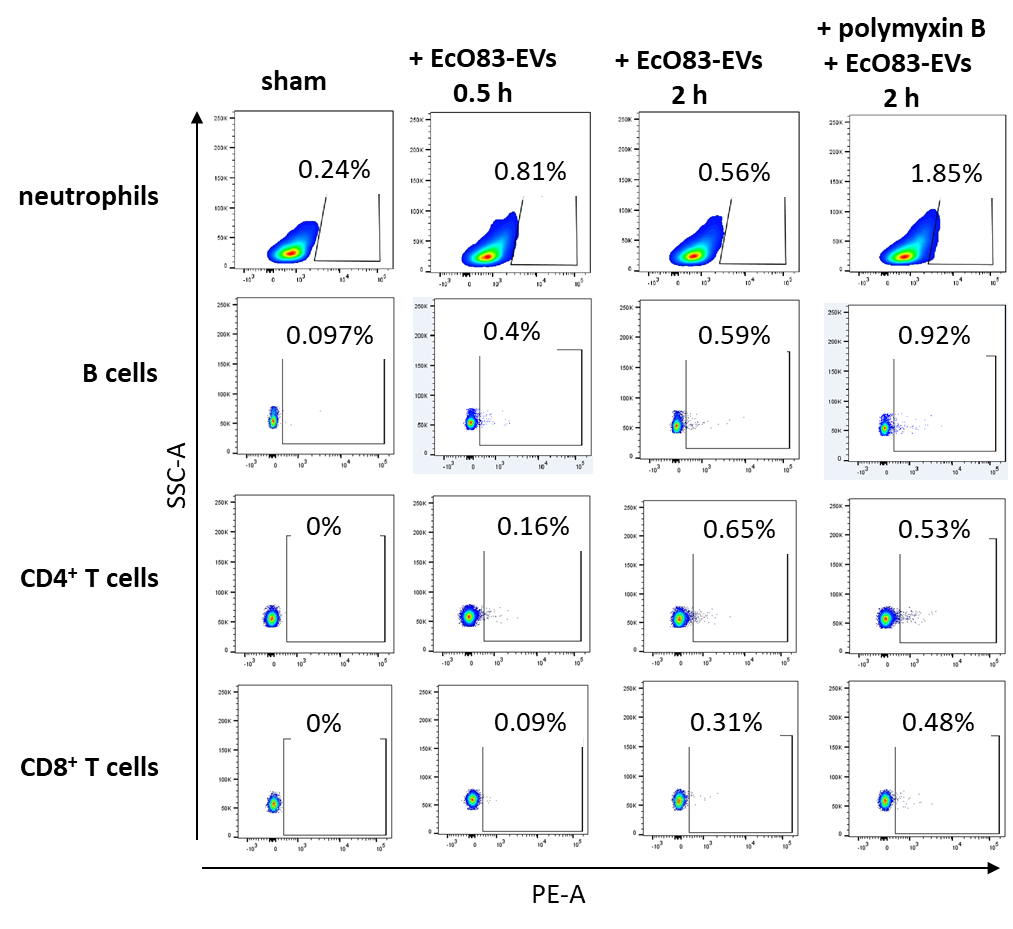
**

**FACS analysis of lung cells isolated from mice treated intranasally with rhodamine-labelled EcO83-EVs.** Mice received 2.5 x 10^10^ rhodamine-labelled EcO83-EVs with/without polymyxin B intranasally and lung samples were collected 0.5 h (n = 5) or 2 h (n = 5) after treatment and analysed by FACS. Control mice (sham; n = 3) received 0.9% NaCl. Data from one representative out of two experiments are shown.

**Figure S7**


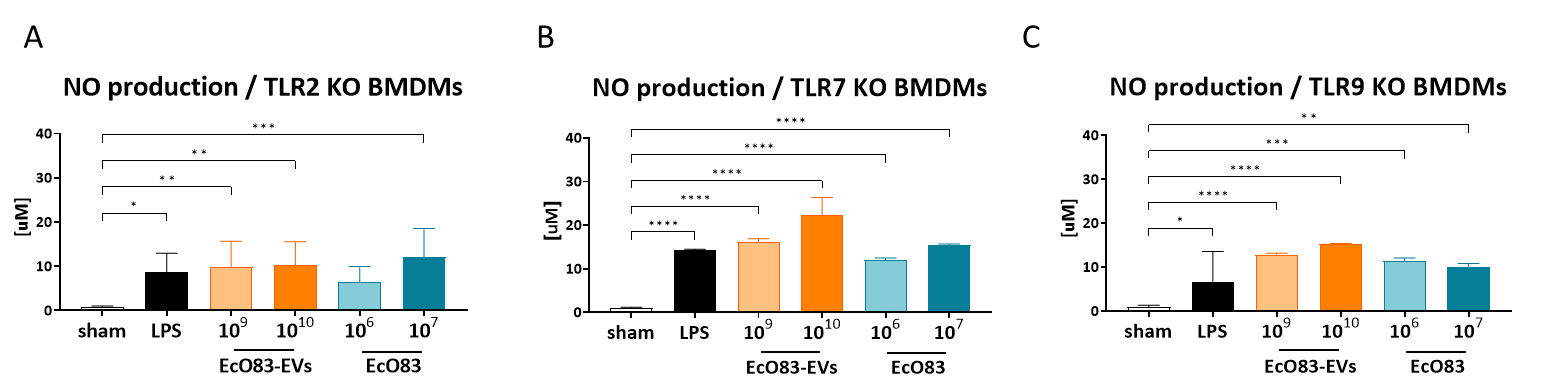


**Production of NO in TLR2, 7 or 9 knockout bone marrow-derived macrophages by EcO83-EVs and EcO83.** TLR2, TLR7, and TLR9 knockout (KO) bone marrow-derived macrophages (BMDMs) were treated with media (sham), 1 μg/ml LPS, EcO83-EV (10^9^ and 10^10^ ) and EcO83 (10^6^ and 10^7^ bacteria/ml) for 24 h. **(A)** NO production in TLR2 KO BMDMs **(B),** NO production in TLR7 KO BMDMs **(C),** NO production in TLR9 KO BMDMs. One-way ANOVA with Dunnett’s multiple comparison test was used to examine mean differences between samples. *p ≤ 0.05, **p ≤ 0.01 ***p≤0.001 ****p≤0.0001 versus control**.**

**Figure S8**


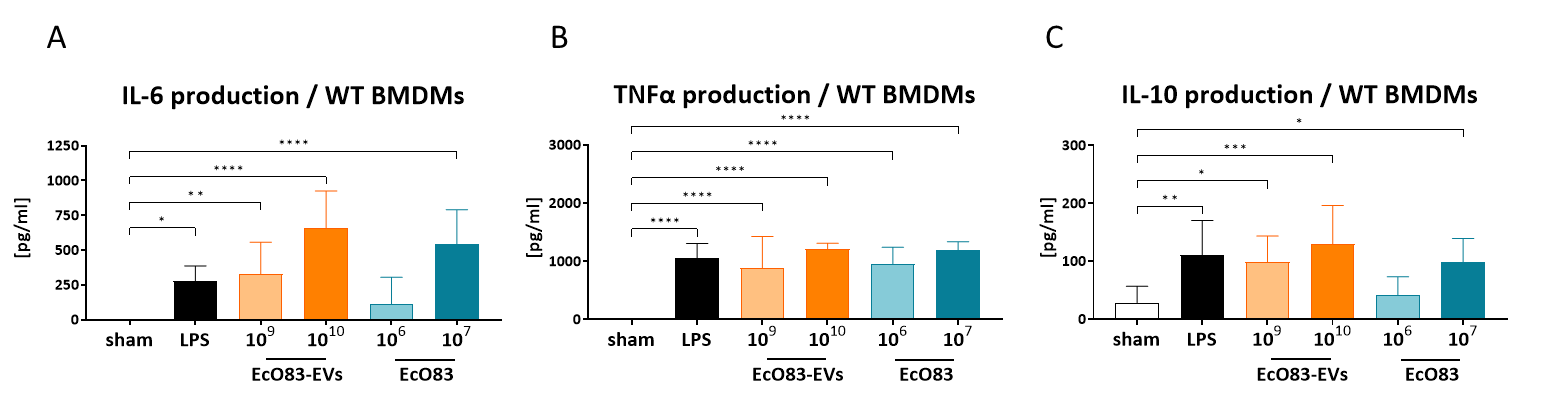


**Production of cytokines in wild type bone marrow-derived macrophages by EcO83-EVs and EcO83.** Wild type (WT) bone marrow-derived macrophages (BMDMs) were treated with media (sham), 1 μg/ml LPS, EcO83-EV (10^9^ and 10^10^ particles/ml) and EcO83 (10^6^ and 10^7^ bacteria/ml) for 24 h. **(A)** Levels of IL-6, **(B)** TNFα and **(C)** IL-10 were measured by ELISA. Results represent at least three independent experiments, and data are presented as mean ± SD. One-way ANOVA with Dunnett’s multiple comparison test used was to examine mean differences between samples. *p ≤ 0.05, **p ≤ 0.01 ***p≤0.001 ****p≤0.0001 versus control**.**

**Figure S9**


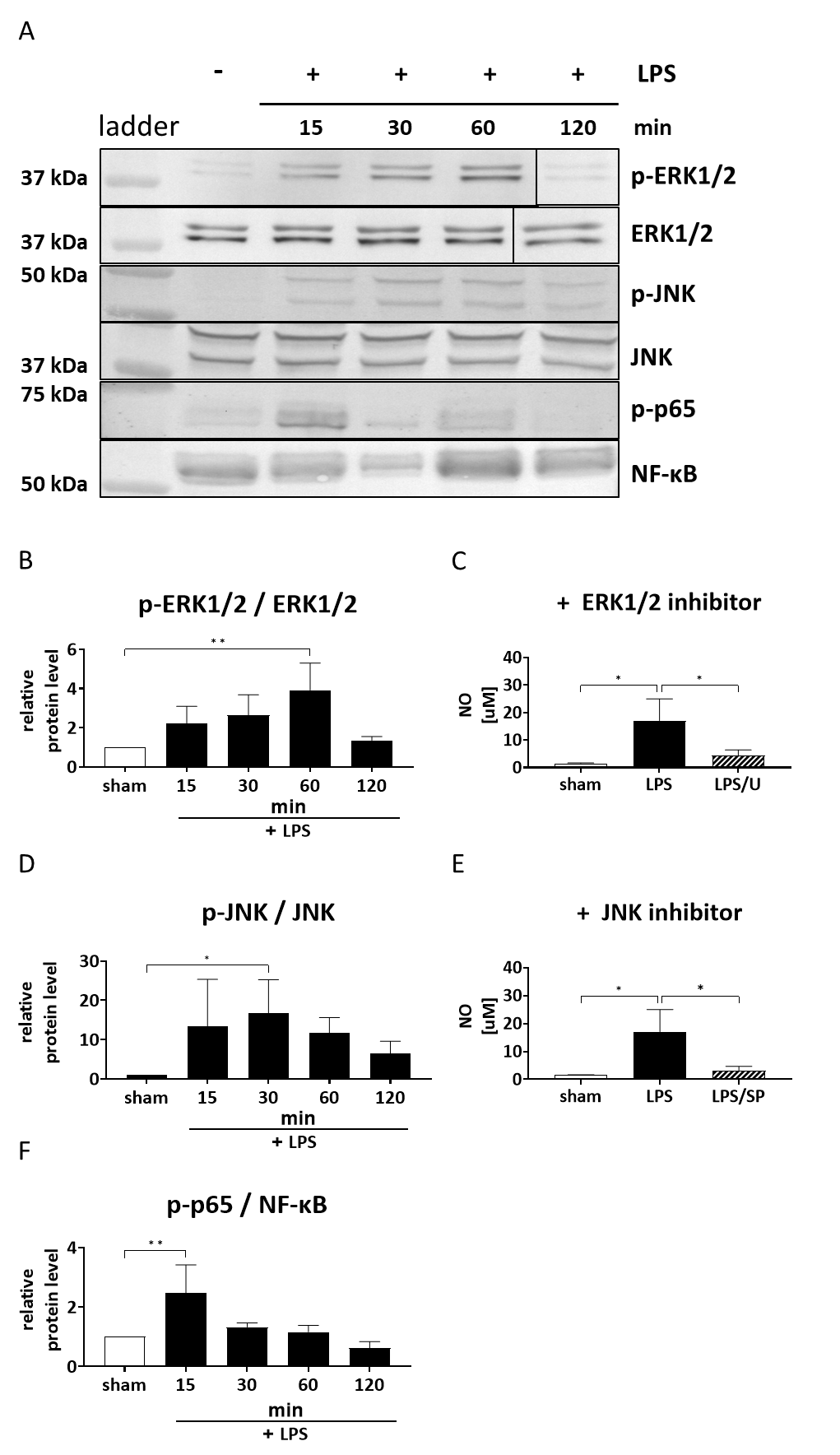


**Effect of LPS from *E. coli* O55B5 on the MAPK and NF-κΒ transcription factor activation in bone marrow-derived macrophages.** Bone marrow-derived macrophages (BMDMs) were stimulated with media (sham) or 1 μg/ml LPS for 15 - 120 min. The level of phosphorylated or non-phosphorylated proteins in the cell lysates was analysed by immunoblotting. **(A)** Phosphorylation status of ERK1/2, JNK and p65 proteins in BMDMs. **(B)** Densitometric analysis of p-ERK1/2 / ERK1/2 in BMDMs. **(C)** Production of NO by BMDMs treated for 24 h with media or 1 μg/ml LPS with/without 1 h pre-treatment with ERK1/2 inhibitor (U0126; 20 μM). **(D)** Densitometric analysis of p-JNK/JNK in BMDM treated with media or 1 μg/ml LPS for 15 - 120 min; **(E)** Production of NO in BMDM treated with media or 1 μg/ml LPS for 24 h with/without 1 h pre-treatment with JNK inhibitor (SP600125; 10 μM). **(F)** Densitometric analysis of p-p65/NFκΒ in BMDMs treated with media or 1 μg/ml LPS for 15 - 120 min. One-way ANOVA with Dunnett’s multiple comparison test was used to analyse the mean differences between the samples. *p ≤ 0.05, **p ≤ 0.01 ***p≤0.001 ****p≤0.0001 versus control**.**

**Table S1. Full list of proteins significantly enriched in EcO83-EVs samples before and after density gradient (DG) purification (cell lysates used as a background).** Flagella and fimbria-related proteins are in bold.

| Proteins enriched in EcO83-EVs sample before DG purification | Proteins that are in both samples – before and after DG purification | Proteins enriched in EcO83-EVs sample after DG purification |
| --- | --- | --- |
| **sp\|P75937\|FLGE_ECOLI** | **sp\|P33235\|FLGK_ECOLI** | sp\|P0AF70\|YJEI_ECOLI |
| **sp\|P0ABX5\|FLGG_ECOLI** | **sp\|P29744\|FLGL_ECOLI** | sp\|P0AES9\|HDEA_ECOLI |
| sp\|P10384\|FADL_ECOLI | sp\|P0A927\|TSX_ECOLI | sp\|P0A937\|BAME_ECOLI |
| **sp\|P0ABW9\|FLGB_ECOLI** | sp\|P13036\|FECA_ECOLI | sp\|P0A7Q1\|RL35_ECOLI |
| **sp\|P0ABX2\|FLGC_ECOLI** | sp\|P77717\|YBAY_ECOLI | **sp\|P08191\|FIMH_ECOLI** |
| sp\|P60785\|LEPA_ECOLI | sp\|P37665\|YIAD_ECOLI | sp\|P0AG59\|RS14_ECOLI |
| **sp\|P52614\|FLIK_ECOLI** | sp\|P10100\|RLPA_ECOLI | sp\|P0ADB4\|ECNA_ECOLI |
| sp\|P0AA16\|OMPR_ECOLI | sp\|P0A921\|PA1_ECOLI | sp\|P64614\|YHCN_ECOLI |
| sp\|P11454\|ENTF_ECOLI | sp\|P0A935\|MLTA_ECOLI | **sp\|P24216\|FLID_ECOLI** |
| sp\|P0ACR9\|MPRA_ECOLI | sp\|P02943\|LAMB_ECOLI | sp\|P75946\|YCFL_ECOLI |
| sp\|P77294\|YDER_ECOLI | sp\|P45464\|LPOA_ECOLI | sp\|P0ABK2\|CYDB_ECOLI |
| sp\|P0A853\|TNAA_ECOLI | sp\|P76115\|YNCD_ECOLI | sp\|Q46835\|YGHG_ECOLI |
| sp\|P0A6R3\|FIS_ECOLI | sp\|P77774\|BAMB_ECOLI | **sp\|P0A6S3\|FLGI_ECOLI** |
| sp\|P0ACJ0\|LRP_ECOLI | sp\|P0ADA7\|OSMB_ECOLI | sp\|P23898\|NLPC_ECOLI |
| sp\|P0ADG4\|SUHB_ECOLI | sp\|P61320\|LOLB_ECOLI | sp\|P0AEL6\|FEPB_ECOLI |
| sp\|P0ACP7\|PURR_ECOLI | sp\|P0C960\|EMTA_ECOLI | sp\|P46122\|YAJI_ECOLI |
| sp\|P0A9G6\|ACEA_ECOLI | sp\|P76206\|YDIY_ECOLI | sp\|P64493\|YOAF_ECOLI |
| **sp\|P75938\|FLGF_ECOLI** | sp\|P0AC02\|BAMD_ECOLI | sp\|P0AF56\|YJCO_ECOLI |
| sp\|P0A6E9\|BIOD2_ECOLI | sp\|Q46798\|YGER_ECOLI | sp\|P0C066\|MLTC_ECOLI |
| sp\|P0A7D1\|PTH_ECOLI | **sp\|P08189\|FIMF_ECOLI** | sp\|P76137\|YDET_ECOLI |
| sp\|P0A887\|UBIE_ECOLI | sp\|P0ADC1\|LPTE_ECOLI | sp\|P08550\|CVPA_ECOLI |
| sp\|P0C058\|IBPB_ECOLI | **sp\|P08190\|FIMG_ECOLI** | sp\|P37028\|BTUF_ECOLI |
| sp\|P69924\|RIR2_ECOLI | sp\|P75780\|FIU_ECOLI | sp\|P0ABV6\|TOLR_ECOLI |
| sp\|P77804\|YDGA_ECOLI | sp\|P65292\|YGDI_ECOLI | sp\|P64499\|YEBO_ECOLI |
| sp\|P0AEE5\|DGAL_ECOLI | sp\|P76506\|MLAA_ECOLI | sp\|P0A7M6\|RL29_ECOLI |
| sp\|P0A8I3\|YAAA_ECOLI | sp\|P0A9E5\|FNR_ECOLI | sp\|P0A6M2\|DSBB_ECOLI |
| sp\|P0CG19\|RNPH_ECOLI | **sp\|P39264\|FIMI_ECOLI** | sp\|P43674\|YCAL_ECOLI |
| sp\|P0C054\|IBPA_ECOLI | sp\|Q7DFV3\|YMGG_ECOLI | sp\|P64448\|YNBE_ECOLI |
| sp\|P0ACP1\|CRA_ECOLI | sp\|P77562\|YAIW_ECOLI | sp\|P25714\|YIDC_ECOLI |
| sp\|P0A734\|MINE_ECOLI | sp\|P0AAB6\|GALF_ECOLI | sp\|P76471\|YFAZ_ECOLI |
| sp\|P21362\|YCIF_ECOLI | sp\|P41052\|MLTB_ECOLI | sp\|P00811\|AMPC_ECOLI |
| sp\|P30958\|MFD_ECOLI | sp\|P46130\|YBHC_ECOLI | sp\|P0AFB1\|NLPI_ECOLI |
| sp\|P46837\|YHGF_ECOLI | **sp\|P0A6S0\|FLGH_ECOLI** | **sp\|P30130\|FIMD_ECOLI** |
| sp\|P64624\|YHEO_ECOLI | sp\|P76537\|YFEY_ECOLI | **sp\|P04128\|FIMA1_ECOLI** |
| sp\|P18196\|MINC_ECOLI | sp\|P0AA91\|YEAY_ECOLI | sp\|Q47534\|YAIO_ECOLI |
| sp\|P09551\|ARGT_ECOLI | sp\|P39180\|AG43_ECOLI | sp\|P75687\|RCLB_ECOLI |
| sp\|P27550\|ACSA_ECOLI | sp\|P0ADN6\|YIFL_ECOLI | sp\|P75820\|AMID_ECOLI |
| **sp\|P04949\|FLIC_ECOLI** | sp\|P63883\|AMIC_ECOLI | sp\|P0ADM4\|YIDQ_ECOLI |
| sp\|P0ACJ8\|CRP_ECOLI | sp\|P37650\|BCSC_ECOLI | sp\|P32719\|ALSE_ECOLI |
| sp\|P0A8F0\|UPP_ECOLI | sp\|P66948\|BEPA_ECOLI | sp\|P31827\|YDDB_ECOLI |
| sp\|P60716\|LIPA_ECOLI | sp\|P31554\|LPTD_ECOLI | sp\|P77348\|MPPA_ECOLI |
| sp\|P0AB89\|PUR8_ECOLI | sp\|P09169\|OMPT_ECOLI | sp\|P64426\|DIGH_ECOLI |
| sp\|P00452\|RIR1_ECOLI | sp\|P0A940\|BAMA_ECOLI | sp\|P16869\|FHUE_ECOLI |
| sp\|P37194\|SLP_ECOLI | sp\|P0A908\|MIPA_ECOLI | sp\|P32160\|YIIQ_ECOLI |
| sp\|P0A7G2\|RBFA_ECOLI | sp\|P06971\|FHUA_ECOLI | sp\|P77789\|YDES_ECOLI |
| sp\|P0A9Q1\|ARCA_ECOLI | sp\|P77330\|BORD_ECOLI | sp\|P0AB26\|YCEB_ECOLI |
| sp\|P60390\|RSMH_ECOLI | sp\|P64596\|YRAP_ECOLI | sp\|P28224\|MLIC_ECOLI |
| sp\|P0A6P7\|ENGB_ECOLI | sp\|P0A912\|PAL_ECOLI | sp\|P77184\|LOMR_ECOLI |
| sp\|P0A7S3\|RS12_ECOLI | sp\|P0A910\|OMPA_ECOLI | sp\|P00803\|LEP_ECOLI |
| sp\|P0A7W1\|RS5_ECOLI | sp\|P0A917\|OMPX_ECOLI | sp\|P39401\|OPGB_ECOLI |
| sp\|P0ABU2\|YCHF_ECOLI | sp\|P21420\|NMPC_ECOLI | sp\|P63235\|GADC_ECOLI |
| sp\|P0AEK2\|FABG_ECOLI | sp\|P0A707\|IF3_ECOLI | sp\|P0AFJ7\|PITA_ECOLI |
| sp\|P0A6H1\|CLPX_ECOLI | sp\|P69776\|LPP_ECOLI | sp\|P0C0T5\|MEPA_ECOLI |
| sp\|P0AB24\|EFEO_ECOLI | sp\|P0ADB1\|OSME_ECOLI | sp\|P0AB12\|YCCF_ECOLI |
| sp\|P0A7J3\|RL10_ECOLI | sp\|P0ADA5\|YAJG_ECOLI | sp\|P29131\|FTSN_ECOLI |
| sp\|P0A9X4\|MREB_ECOLI | sp\|P0A915\|OMPW_ECOLI | sp\|P13656\|CHIA_ECOLI |
| sp\|P02413\|RL15_ECOLI | sp\|P76513\|YFDQ_ECOLI | sp\|P36548\|AMIA_ECOLI |
| sp\|P37634\|RLMJ_ECOLI | sp\|P0A905\|SLYB_ECOLI | sp\|P11289\|YFIL_ECOLI |
|  | sp\|P0A903\|BAMC_ECOLI | sp\|P33343\|YEHD_ECOLI |
|  | sp\|P0A7G6\|RECA_ECOLI | sp\|P25894\|LOIP_ECOLI |
|  | sp\|P0ADA3\|NLPD_ECOLI | sp\|P0A9P6\|DEAD_ECOLI |
|  | sp\|P0A7M2\|RL28_ECOLI | sp\|P0AEQ6\|GLNP_ECOLI |
|  | sp\|P0AAX8\|YBIS_ECOLI | sp\|P32681\|YJAH_ECOLI |
|  | sp\|P68688\|GLRX1_ECOLI | sp\|P00393\|NDH_ECOLI |
|  | sp\|P17315\|CIRA_ECOLI | sp\|P31545\|EFEB_ECOLI |
|  | sp\|P0A7V3\|RS3_ECOLI | sp\|P65290\|YFGH_ECOLI |
|  | sp\|P0ADY7\|RL16_ECOLI | sp\|P28631\|HOLB_ECOLI |
|  | sp\|P0A7V0\|RS2_ECOLI | sp\|P39165\|YCHO_ECOLI |
|  | sp\|P0A7R9\|RS11_ECOLI | sp\|P0AAD6\|SDAC_ECOLI |
|  | sp\|P0A7K6\|RL19_ECOLI | sp\|P03841\|MALM_ECOLI |
|  | sp\|P61175\|RL22_ECOLI | **sp\|P75933\|FLGA_ECOLI** |
|  | sp\|P76177\|YDGH_ECOLI | sp\|P0ABU7\|EXBB_ECOLI |
|  | sp\|P0A7T3\|RS16_ECOLI | sp\|P46022\|MTGA_ECOLI |
|  | sp\|P0A7J7\|RL11_ECOLI | sp\|P75954\|YCFS_ECOLI |
|  | sp\|P0AEE1\|DCRB_ECOLI | sp\|P0DMC7\|RCSB_ECOLI |
|  | sp\|P0A7U7\|RS20_ECOLI | sp\|P60752\|MSBA_ECOLI |
|  | sp\|P0A855\|TOLB_ECOLI | sp\|P76223\|YNJB_ECOLI |
|  | sp\|P45955\|CPOB_ECOLI | sp\|Q46863\|YGIS_ECOLI |
|  | sp\|P0DTT0\|BIPA_ECOLI | sp\|P0AC30\|FTSX_ECOLI |
|  | sp\|P0AG55\|RL6_ECOLI | sp\|P10903\|NARK_ECOLI |
|  | sp\|P06996\|OMPC_ECOLI | sp\|P42632\|TDCE_ECOLI |
|  |  | sp\|P0AB10\|PQIC_ECOLI |
|  |  | sp\|P23865\|PRC_ECOLI |
|  |  | sp\|P76249\|LEUE_ECOLI |
|  |  | sp\|P0AA99\|YAFK_ECOLI |
|  |  | sp\|P0AAJ8\|HYBA_ECOLI |
|  |  | sp\|P0A9N4\|PFLA_ECOLI |
|  |  | sp\|P0ABK9\|NRFA_ECOLI |
|  |  | sp\|P25737\|LYSP_ECOLI |
|  |  | sp\|P0AC75\|KDTA_ECOLI |
|  |  | sp\|P0AD44\|YFHG_ECOLI |
|  |  | sp\|P15028\|FECB_ECOLI |
|  |  | sp\|P0ADT2\|YGIB_ECOLI |
|  |  | sp\|P14175\|PROV_ECOLI |
|  |  | sp\|P0A843\|TATE_ECOLI |
|  |  | sp\|P0AEH1\|RSEP_ECOLI |
|  |  | sp\|P0AEX9\|MALE_ECOLI |
|  |  | sp\|P37636\|MDTE_ECOLI |
|  |  | sp\|P05825\|FEPA_ECOLI |
|  |  | sp\|P0ABJ1\|CYOA_ECOLI |
|  |  | sp\|P0A6P5\|DER_ECOLI |
|  |  | sp\|P77338\|MSCK_ECOLI |
|  |  | sp\|P06129\|BTUB_ECOLI |
|  |  | sp\|P02931\|OMPF_ECOLI |
|  |  | sp\|P40120\|OPGD_ECOLI |
|  |  | sp\|P69411\|RCSF_ECOLI |
|  |  | sp\|P0AG63\|RS17_ECOLI |
|  |  | sp\|P0ADZ0\|RL23_ECOLI |
|  |  | sp\|P0AGC3\|SLT_ECOLI |
|  |  | sp\|P0AD59\|IVY_ECOLI |
|  |  | sp\|P0ADE4\|TAMA_ECOLI |
|  |  | sp\|P23331\|KITH_ECOLI |
|  |  | sp\|P21365\|YCIC_ECOLI |
|  |  | sp\|P42604\|UXAA_ECOLI |
|  |  | sp\|P25736\|END1_ECOLI |
|  |  | sp\|P0AET2\|HDEB_ECOLI |
|  |  | sp\|P60422\|RL2_ECOLI |
|  |  | sp\|P0AEG6\|DSBC_ECOLI |
|  |  | sp\|P18390\|YJJA_ECOLI |
|  |  | sp\|P0AES6\|GYRB_ECOLI |
|  |  | sp\|P40710\|NLPE_ECOLI |
|  |  | sp\|P31063\|YEDD_ECOLI |
|  |  | sp\|P65294\|YGDR_ECOLI |
|  |  | sp\|P64451\|YDCL_ECOLI |
|  |  | sp\|P0AFL3\|PPIA_ECOLI |
|  |  | sp\|P0ADY3\|RL14_ECOLI |
|  |  | sp\|P0ADB7\|ECNB_ECOLI |
|  |  | sp\|P75830\|MACA_ECOLI |
|  |  | sp\|P04968\|ILVA_ECOLI |
|  |  | sp\|P00550\|PTM3C_ECOLI |
|  |  | sp\|P75818\|YBJP_ECOLI |
|  |  | sp\|P76445\|LPXT_ECOLI |
|  |  | sp\|P0AFM2\|PROX_ECOLI |
|  |  | sp\|P0AG78\|SUBI_ECOLI |
|  |  | sp\|P60723\|RL4_ECOLI |
|  |  | sp\|P0A9B6\|E4PD_ECOLI |
|  |  | sp\|P0A7T7\|RS18_ECOLI |
|  |  | sp\|P21179\|CATE_ECOLI |
|  |  | sp\|P15877\|DHG_ECOLI |
|  |  | sp\|P0ADZ4\|RS15_ECOLI |
|  |  | sp\|P0C0L7\|PROP_ECOLI |
|  |  | sp\|P02930\|TOLC_ECOLI |
|  |  | sp\|P0AG44\|RL17_ECOLI |
|  |  | **sp\|P31697\|FIMC_ECOLI** |
|  |  | sp\|P37648\|YHJJ_ECOLI |
|  |  | sp\|P0ABI8\|CYOB_ECOLI |
|  |  | sp\|P42616\|YQJC_ECOLI |
|  |  | sp\|P22523\|MUKB_ECOLI |
|  |  | sp\|P33136\|OPGG_ECOLI |
|  |  | sp\|P08506\|DACC_ECOLI |
|  |  | sp\|P16700\|CYSP_ECOLI |
|  |  | sp\|P0A7S9\|RS13_ECOLI |
|  |  | sp\|P76002\|PLIG_ECOLI |
|  |  | sp\|P61316\|LOLA_ECOLI |
|  |  | sp\|P0AB46\|YMGD_ECOLI |
|  |  | sp\|P62399\|RL5_ECOLI |
|  |  | sp\|P0AA10\|RL13_ECOLI |
|  |  | sp\|P0A993\|F16PA_ECOLI |
|  |  | sp\|P78067\|YNJE_ECOLI |
|  |  | sp\|P0ABZ6\|SURA_ECOLI |
|  |  | sp\|P33590\|NIKA_ECOLI |
|  |  | sp\|P02925\|RBSB_ECOLI |
|  |  | sp\|P27434\|RODZ_ECOLI |
|  |  | sp\|P77588\|YDEQ_ECOLI |
|  |  | sp\|P0A7L3\|RL20_ECOLI |
|  |  | sp\|P0AEB2\|DACA_ECOLI |
|  |  | sp\|P0AFK9\|POTD_ECOLI |
|  |  | sp\|P0ABJ9\|CYDA_ECOLI |
|  |  | sp\|P0AE91\|CREA_ECOLI |
|  |  | sp\|P76116\|YNCE_ECOLI |
|  |  | sp\|P0ABL3\|NAPB_ECOLI |
|  |  | sp\|P0AEU7\|SKP_ECOLI |
|  |  | sp\|P0ADV7\|MLAC_ECOLI |
|  |  | sp\|P0C018\|RL18_ECOLI |
|  |  | sp\|P0AB55\|YCII_ECOLI |
|  |  | sp\|P45523\|FKBA_ECOLI |
|  |  | sp\|P0A7R5\|RS10_ECOLI |
|  |  | sp\|P23894\|HTPX_ECOLI |
|  |  | sp\|P11557\|DAMX_ECOLI |
|  |  | sp\|P0A7E1\|PYRD_ECOLI |
|  |  | sp\|P0A7V8\|RS4_ECOLI |
|  |  | sp\|P0AEQ3\|GLNH_ECOLI |
|  |  | sp\|P19926\|AGP_ECOLI |
|  |  | sp\|P08331\|CPDB_ECOLI |
|  |  | sp\|P0ADU5\|YGIW_ECOLI |
|  |  | sp\|P30859\|ARTI_ECOLI |
|  |  | sp\|P24182\|ACCC_ECOLI |
|  |  | sp\|P28635\|METQ_ECOLI |
|  |  | sp\|P05458\|PTRA_ECOLI |
|  |  | sp\|P0C0S1\|MSCS_ECOLI |
|  |  | sp\|P0AEM9\|TCYJ_ECOLI |
|  |  | sp\|P0ADS9\|YGGN_ECOLI |
|  |  | sp\|P0A7X3\|RS9_ECOLI |
